# The Role of Individual Characteristics in Predicting Short- and Long-Term Cognitive and Psychological Benefits of Cognitive Stimulation Therapy for Mild-to-Moderate Dementia

**DOI:** 10.3389/fnagi.2021.811127

**Published:** 2022-01-11

**Authors:** Elena Carbone, Federica Piras, Massimiliano Pastore, Erika Borella

**Affiliations:** ^1^Department of General Psychology, University of Padova, Padua, Italy; ^2^Neuropsychiatry Laboratory, Clinical and Behavioral Neurology Department, Istituto di Ricovero e Cura a Carattere Scientifico (IRCCS) Santa Lucia Foundation, Rome, Italy; ^3^Department of Developmental Psychology and Socialization, University of Padova, Padua, Italy

**Keywords:** cognitive stimulation therapy, dementia, cognitive functioning, depression, behavioral and neuropsychiatric, quality of life, individual differences

## Abstract

**Introduction:** This study examined the role of individual characteristics in predicting short- and long-term benefits of the Italian version of Cognitive Stimulation Therapy (CST-IT), an evidence-based intervention for people with mild-to-moderate dementia.

**Materials and Methods:** Data were drawn from a sample (*N* = 123) of people with dementia (PwD) who took part in a multicenter controlled clinical trial of CST-IT. Assessments at pre-test, immediately after completing the treatment, and 3 months later investigated the following outcomes: general cognitive functioning and language, mood and behavior, everyday functioning, and quality of life. Age, education and baseline (pre-test) cognitive functioning, mood (depression) and behavioral and neuropsychiatric symptoms were considered as predictors of any short- and long-term benefits.

**Results:** Linear mixed-effects models showed that different individual characteristics -particularly education and age- influenced the benefits of CST-IT, depending on the outcome measures considered. Higher education predicted larger gains in general cognitive functioning and, along with less severe depressive symptoms, in language (magnification effects). Older age was associated with positive changes in mood (compensation effects). Albeit very modestly, older age was also associated with larger gains in everyday functioning (compensation effects). Gains in quality of life were predicted by older age and lower education (compensation effects). Baseline cognitive functioning, mood and/or behavioral symptoms broadly influenced performance too, but their role again depended on the outcomes considered.

**Discussion:** These findings underscore the importance of considering and further exploring how psychosocial interventions like CST are affected by individual characteristics in order to maximize their efficacy for PwD.

## Introduction

Dementia is a neurocognitive disorder caused by neurodegeneration of various etiologies (American Psychiatric Association, 2013). People with dementia (PwD) have to cope with gradually worsening impairments in multiple cognitive domains, marked changes in their emotional, social and behavioral control, and declining abilities in activities of daily living (American Psychiatric Association, 2013). It has thus become a priority to sustain their residual cognitive and relational abilities, and contain their behavioral issues in an effort to improve patients’ and their caregivers’ quality of life (Woods et al., 2012; McDermott et al., 2018).

While researchers are still struggling to find disease-modifying pharmacological therapies to slow the progression of dementia, psychosocial (non-pharmacological) approaches based on cognitive stimulation (CS) -offering a range of enjoyable activities and broadly stimulating the individual’s thinking, concentration and memory- have received increasing attention in recent years (Woods et al., 2012; McDermott et al., 2018).

One such CS program is the evidence-based Cognitive Stimulation Therapy (CST; Spector et al., 2003, 2006), which combines effective elements of other CS programs (reality orientation, reminiscence, multisensory stimulation, implicit learning) with a person-centered approach. CST promotes the engagement of PwD in a variety of enjoyable group activities to stimulate various cognitive abilities (i.e., language and executive functioning, spatial and temporal orientation, reminiscence, and retrieval of personal information), and their emotional, relational and social skills (Woods et al., 2012). CST has produced benefits—immediately after its completion, at least—in terms of general cognitive functioning, language comprehension and narrative abilities (see Lobbia et al., 2019 for a review). The program also has a positive impact on other dementia-related symptoms (e.g., behavioral disorders, depression), and quality of life (Piras et al., 2017; Chao et al., 2020). A recent multicenter randomized controlled trial (RCT; Carbone et al., 2021) involving 255 PwD at 16 residential care homes and day centers all over Italy examined the efficacy of the Italian (IT) adaptation of the original CST protocol (CST-IT; Capotosto et al., 2017) immediately and then 3 months after completing the treatment. This RCT confirmed that CST-IT is effective (compared with a treatment-as-usual active control condition) in supporting cognitive and emotional functioning, and counteracting the progression of behavioral/neuropsychiatric symptoms in PwD. The RCT was also the first to demonstrate that these benefits persist over time (about 5 months after starting the treatment).

Despite growing evidence of the efficacy of CST for PwD, it remains unclear whether and to what extent different individual characteristics might influence its benefits. One study found older age associated with greater gains in general cognitive functioning after CST (Aguirre et al., 2013). Another (Apoìstolo et al., 2014) found no association between baseline functional dependence (measured with the Barthel Activities of Daily Living index) and the benefits of CST on cognition and mood. Two other recent studies examined whether CST benefited PwD differently depending on the severity of their dementia (Marinho et al., 2020), and their estimated cognitive reserve [i.e., their lifetime exposure to mentally engaging activities that promote more efficient brain networks and processes when performing tasks (Stern, 2002, 2021)]. Participants’ cognitive reserve was operationalized in terms of years of formal education (Marinho et al., 2020) or level of involvement (low-medium vs. high) in cognitively-stimulating educational, occupational and leisure activities (Alvares Pereira et al., 2020). Both studies found no impact of the factors explored on the benefits of CST, however, going against expectations that participants’ cognitive reserve would affect their brain plasticity, and capacity to engage compensatory processing mechanisms (Stern, 2002, 2021; Barulli and Stern, 2013).

Beyond the scant evidence on individual factors likely to influence the short-term benefits of CST, whether predictors of such gains influence longer-term results remains to be seen. It is essential to understand whether and to what extent PwD with different individual profiles might benefit from the treatment, and which outcomes will be the most affected, in order to maximize the protocol’s efficacy in clinical practice.

The present study therefore aimed to more comprehensively examine how different individual characteristics might predict short- and long-term benefits of CST-IT in: general cognitive functioning and language (primary outcome measures); mood and behavior/neuropsychiatric symptoms; everyday life functioning; and quality of life (secondary outcome measures). The individual characteristics considered for their potential influence on the benefits of CST were: (i) socio-demographic characteristics (i.e., age and education); (ii) baseline general cognitive functioning, as measured by the Mini-Mental State Examination (MMSE; Folstein et al., 1975), because the complexity of the activities proposed in CST sessions depends on participants’ baseline general cognitive functioning scores (see Carbone et al., 2021 for further details); and (iii) baseline mood (depressive symptoms) and behavioral/neuropsychiatric symptoms of dementia, given the interactions between cognitive, mood and behavioral symptoms in mild-to-moderate dementia (Dillon et al., 2013), and the neurobiological link between mood disturbances, neurodegeneration and the emergence/progression of cognitive impairment (e.g., Köhler et al., 2015; Andrews et al., 2018).

According to the compensation and magnification hypotheses (Lövdén et al., 2012), the clinical benefits of CST might be more evident in older, less educated, and more cognitively impaired PwD than in younger, more educated PwD with an apparently better-preserved cognitive profile (compensation effect). This is because CST might provide the former with an “enriched” and stimulating environment capable of re-activating and supporting their residual skills. It could be less beneficial in less-impaired PwD with more efficient functional and behavioral task-performing processes, who are still relying on compensatory processing mechanisms. The magnification hypothesis would envisage the opposite pattern of results. Younger, better educated and more cognitively preserved PwD could benefit more from CST because their residual resources would enable them to engage more fully in the CST sessions (magnification effect). Mood (depression), behavioral and neuropsychiatric symptoms (e.g., apathy, delirium, agitation) could limit the compliance of PwD with any treatment and their participation in stimulating activities, resulting in poor rehabilitation outcomes (e.g., Williams, 2005; Buettner et al., 2011). On the other hand, more engaging and motivating exercises might arouse apathetic patients’ interest (Manera et al., 2015), making the presence of an affective/motivation disorder a significant predictor of a better outcome of CST. All that said, whether compensatory or magnifying effects can be expected from CST in participants who have mood issues, or baseline behavioral or neuropsychiatric symptoms has yet to be explored.

Compensatory and magnifying effects relating to individual characteristics are not mutually exclusive [they can emerge differently after CS interventions, depending on the outcome measure considered (Borella et al., 2017)], so different predictors of gains would presumably account for these compensatory or magnifying effects after a CST program too, again depending on the variable considered.

## Methods

### Participants

Data on 123 people with mild-to-moderate dementia forming the CST-IT group in a previous single-blind (assessor-blinded), multicenter, controlled clinical trial were examined. Major eligibility criteria were (e.g., Spector et al., 2003): (a) a diagnosis of major neurocognitive disorder (of any etiological subtype) according to the fifth edition of the Diagnostic and Statistical Manual of Mental Disorders, in the mild-to-moderate range (MMSE score at least above 14)^1^; (b) a Clinical Dementia Rating (Hughes et al., 1982) score of 1 or 2; (c) a satisfactory ability to understand and communicate (for further details on the inclusion/exclusion criteria, see Carbone et al., 2021). Supplementary Table 1 shows the descriptive statistics for participants’ demographics and baseline MMSE scores.

### Materials

For the primary outcomes, the *Alzheimer’s Disease Assessment Scale—Cognitive subscale* (ADAS-Cog; Rosen et al., 1984) was used to measure general cognitive functioning, and the *Narrative Language Test* (NLT; Carlomagno et al., 2013) was administered to assess participants’ narrative abilities in terms of their effectiveness in communicating relevant information. For the secondary outcomes, the *Cornell scale* (Alexopoulos et al., 1988) was used to assess depressive symptoms, and the *Neuropsychiatric Inventory* (NPI; Cummings et al., 1994) to ascertain the frequency and severity of behavioral and neuropsychiatric symptoms. The *Disability Assessment for Dementia* (DAD; De Vreese et al., 2008) was adopted as a measure of everyday functioning, and the *Quality of Life—Alzheimer’s Disease scale* (QoL-AD; Logsdon et al., 1999) was used to assess quality of life (for a detailed explanation, see Supplementary Materials---Description of the outcomes).^2^

### Procedure

All participants attended 20 sessions of CST over a period of 23 weeks. Six were individual sessions for the pre-test, post-test and follow-up assessments. The other 14 were group sessions, scheduled twice a week for 7 weeks, during which the CST-IT was administered (for further details regarding the procedure, see Carbone et al., 2021).

### Statistical Analyses

Bayesian mixed-effects models were run for each measure of interest, with Assessment session (pre-test vs. post-test vs. follow-up), age, education and baseline (pre-test) scores on the MMSE, Cornell scale and NPI as predictors. Subjects and residential care homes (centers) were considered as random effects.

The models were fitted using a fully Bayesian approach with the MCMC (Markov Chain Monte Carlo) estimation method implemented in STAN (Stan Development Team, 2019), using the brms package in R (Bürkner, 2017, 2018). Weak informative priors were used for the regression coefficients, and brms default priors were settled for intercepts, standard deviations (of random effects and residuals), and correlations (for further details, see Supplementary Materials—2.1. Choice of priors, and 2.2. Estimation details).

A model comparison strategy was used first to identify the best model for each outcome measure, based on the following indices: the Leave-One-Out cross-validation Information Criterion (LOO; Vehtari et al., 2017) the Bayesian *R*^2^ (Gelman et al., 2019), and the model weights (*w*; Yao et al., 2018). Lower values of LOO, and higher values of *w* indicate a more plausible model. Then posterior distributions of each best model, i.e., the model with the highest *w*, were analyzed (for further details, see Supplementary Materials—2.3. Model comparisons, and 3. Results).

## Results

Supplementary Table 1 shows the descriptive statistics for each measure of interest, by assessment session. A summary of the results is given in Table 1.

**TABLE 1:** Summary of the assessment session X individual characteristics interactions, and main effects for each of the outcome measures considered, with the % of variance explained by predictors and random effects (subjects and centers).

	Predictors					% of variance explained by	
Outcome	Age	Education	Baseline MMSE	Baseline Cornell	Baseline NPI	Predictors	Random effects
ADAS-Cog		✓ (magn)	*	∼	∼	33%	54%
NLT		✓ (magn)	*	✓ (magn)	∼	25%	55%
Cornell	✓ (comp)				*	38%	44%
NPI	∼	∼		∼		35%	54%
DAD	✓ (comp)			*	*	12%	79%
QoL-AD	✓ (comp)	✓ (comp)	*		*	7%	87%

*ADAS-Cog, Alzheimer’s Disease Assessment Scale—Cognitive subscale; NLT, Narrative Language Test; Cornell, Cornell scale; NPI, NeuroPsychiatric Inventory; DAD, Disability Assessment for Dementia; QoL-AD, Quality of Life—Alzheimer’s Disease scale; MMSE: Mini-Mental State Examination.*

*✓Individual characteristics that showed an interaction with Assessment Session (pre-test, post-test, follow-up); magn, magnifying effect; comp, compensatory effect.*

*Main effects. ∼Main effects showing a modest contribution or marked uncertainty.

### Primary Outcome Measures

#### General Cognitive Functioning

For the ADAS-Cog, the best model was given by the Education X Assessment session interaction, with the baseline Cornell, MMSE and NPI scores as main effects, with random subject and center intercepts. The model was about 2 times more plausible than the next one (see Supplementary Materials—3.1.1. ADAS-Cog), with fixed effects explaining about 33%, and random effects explaining another 54% of the variance in ADAS-Cog scores (*R*^2^ = 0.88).

**FIGURE 1:**
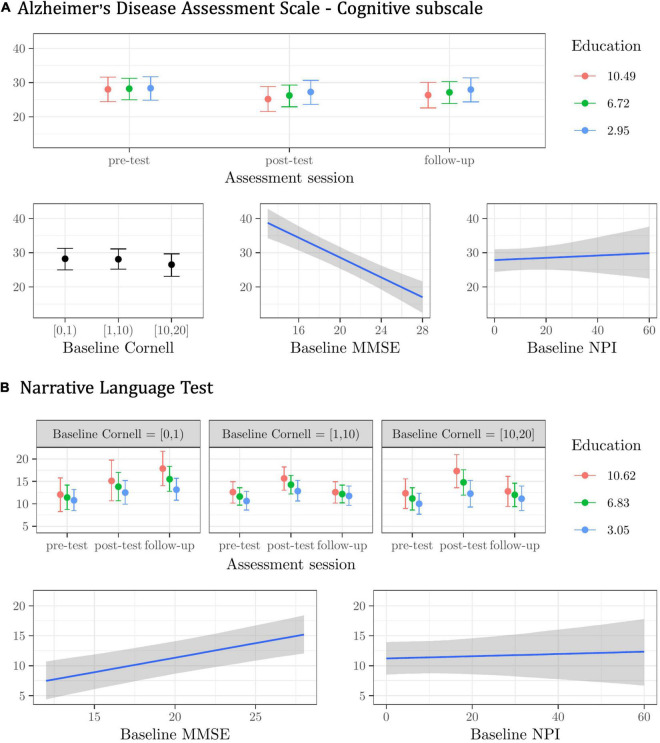
Plots of the best model’s predictions for the primary outcome measures. **(A)** Alzheimer’s Disease Assessment Scale—Cognitive subscale (ADAS-Cog). **(B)** Narrative Language Test (NLT). For each measure, the plots represent the conditional effects of the predictors on the dependent variable.

The (albeit modest) interaction effect indicated that participants with a higher education scored lower on the ADAS-Cog (i.e., their general cognitive functioning improved) from pre-test to post-test, and seemed to retain this gain at follow-up (see Figure 1A). Regardless of assessment session and other predictors, participants scoring higher on the MMSE at the baseline scored lower (i.e., fared better) on the ADAS-Cog. Higher Cornell scores at the baseline were predictive (though only modestly) of lower ADAS-Cog scores. The contribution of baseline NPI scores in predicting general cognitive performance was very modest, and the model’s predictions for higher scores in the NPI were very uncertain (see Figure 1A).

#### Language

For the NLT, the best model included the baseline Cornell X Education X Assessment session interaction, with the baseline MMSE and NPI scores as main effects, and random subject and center intercepts. This model was about one time more plausible than the next one (see Supplementary Materials—3.1.2. NLT), with fixed effects explaining about 25%, and random effects explaining another 55% of the variance in the NLT scores (*R*^2^ = 0.80). Participants with a higher education and mild or no depressive symptoms at the baseline improved the most in their NLT performance from pre-test to post-test and follow-up (see Figure 1B). Regardless of assessment session and other predictors, participants scoring higher on the baseline MMSE performed better in the NLT (see Figure 1B). The contribution of the baseline NPI scores in predicting test performance was very modest, and the model’s predictions for higher scores in the NPI were highly uncertain (see Figure 1B).

### Secondary Outcome Measures

#### Mood

For the Cornell scale, the best model was given by the Age X Assessment session interaction, with the baseline NPI scores as a main effect, and random subject and center intercepts. The model was about one time more plausible than the next one (see Supplementary Materials—3.2.1 Cornell scale), with fixed effects explaining about 38%, and random effects explaining another 44% of the variance in the Cornell scale scores (*R*^2^ = 0.82).

**FIGURE 2:**
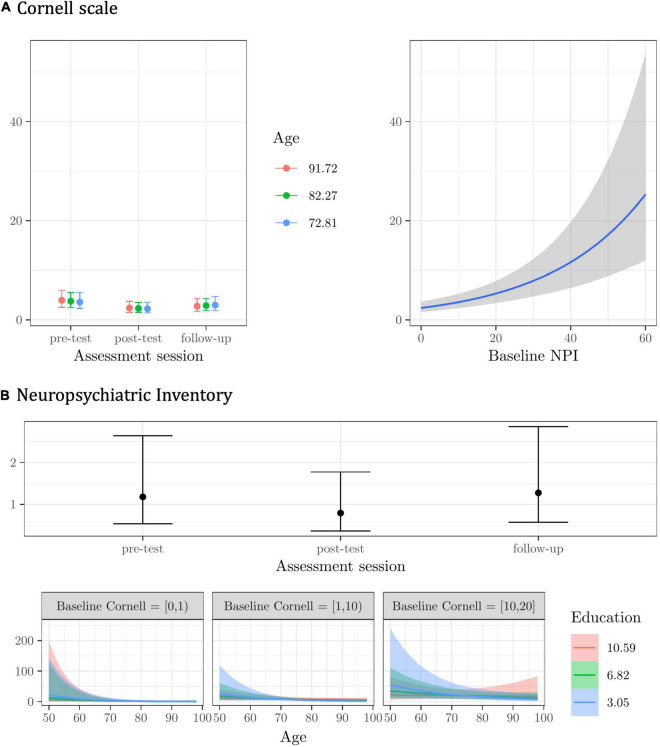
Plots of the best model’s predictions for the secondary outcome measures. **(A)** Cornell scale -depression symptoms-. **(B)** Neuropsychiatric Inventory (NPI) -behavioral/neuropsychiatric symptoms-. For each measure, the plots represent the conditional effects of the predictors on the dependent variable.

The modest effect of the Age X Assessment session interaction suggests that it was the older participants whose scores on the Cornell scale decreased the most from pre-test to post-test, and this gain seemed to persist at follow-up (see Figure 2A). Regardless of assessment session and other predictors, participants scoring higher on the NPI at the baseline scored higher on the Cornell scale (see Figure 2A).

#### Behavior

For the NPI, the best model was given by the Age X baseline Cornell X Education interaction, with Assessment session as a main effect, and random subject and center intercepts. The model was about one time more plausible than the next one (see Supplementary Materials—3.2.2. NPI), with fixed effects explaining about 35% and random effects another 54% of the variance in NPI scores (*R*^2^ = 0.90).

Participants reported less frequent and less severe behavioral and neuropsychiatric symptoms right after completing the CST-IT (at post-test), but this improvement was not maintained at follow-up (see Figure 2B). NPI scores seemed to differ by education level among younger participants with higher baseline scores on the Cornell scale (i.e., with a probable/definite major depressive disorder), but the model’s predictions were very uncertain (see Figure 2B).

#### Everyday Functioning

For the DAD, the best model included the Age X Assessment session interaction, with the baseline Cornell and NPI scores as main effects, and random subject and center intercepts. The model was about two times more plausible than the next one (see Supplementary Materials—3.3.1 DAD). Fixed effects explained only about 12% of the variance in the DAD scores, whereas random effects explained another 79% (*R*^2^ = 0.91).

**FIGURE 3:**
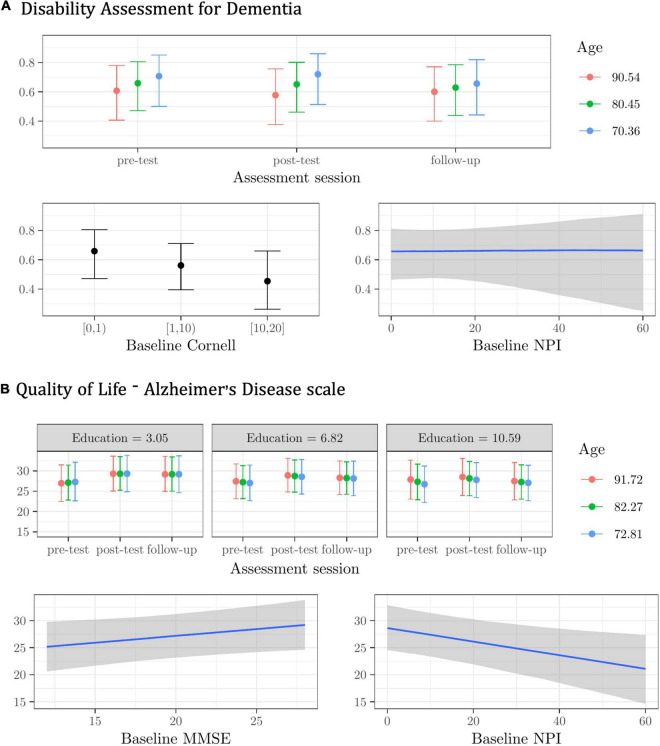
Plots of the best model’s predictions for the secondary outcome measures. **(A)** Disability Assessment for Dementia (DAD). **(B)** Quality of Life-Alzheimer’s Disease scale (QoL-AD). For each measure, the plots represent the conditional effects of the predictors on the dependent variable.

Younger individuals had lower DAD scores at follow-up (see Figure 3A). Regardless of assessment session and other predictors, participants with higher Cornell and NPI scores at the baseline had lower scores in the DAD (see Figure 3A).

#### Quality of Life

For the QoL-AD, the best model was given by the Age X Education X Assessment session interaction, with the baseline MMSE and NPI as main effects, and random subject and center intercepts. This model was only about one time more plausible than the next one, however (see Supplementary Materials—3.3.2 QoL-AD). Fixed effects explained only about 7% of the variance in the QoL-AD scores, whereas random effects explained another 87% (*R*^2^ = 0.94).

The very modest Age X Education X Assessment session interaction effect suggests that less-educated older participants benefited slightly more from the CST-IT in terms of perceived quality of life at post-test and follow-up. Regardless of assessment session and other predictors, participants scoring higher on the baseline MMSE scored higher in the QoL-AD, whereas those with higher scores in the NPI scored lower in the QoL-AD (see Figure 3B).

## Discussion

There is a growing body of evidence of the benefits of CST, also in the Italian version (CST-IT), on cognitive and emotional/behavioral functioning, and quality of life in people with mild-to-moderate dementia. The present study explored whether and to what extent individual characteristics of PwD might predict the cognitive, behavioral, and psychological benefits of CST-IT in the short and longer term. To our knowledge at least, this is the first study to have thoroughly examined this issue, and to have also explored the potential determinants of long-term effects of CST, analyzing data from an RCT (Carbone et al., 2021).

Concerning the primary outcomes (cognition), our better-educated PwD showed greater short- and longer-term benefits of CST-IT in terms of general cognitive functioning (ADAS-Cog scores) and narrative abilities (NLT scores). Education is considered a proxy of cognitive reserve (CR; Stern, 2002, 2021; Barulli and Stern, 2013), so these results point to a role for CR in magnifying the cognitive benefits of CST for PwD: better-educated participants could gain more from CST in the short and longer term thanks to their residual compensatory processing abilities and resources. More efficient brain networks and task-performing processes, which are presumably associated with a greater CR (Stern, 2002, 2021; Barulli and Stern, 2013), could thus influence the cognitive benefits of CST. A synergism also emerged between CR and baseline mood, and accounted for the gains induced by CST-IT in participants’ language and communication skills. Among the best-educated individuals, those with mild or no baseline depressive symptoms improved the most in their NLT scores from pre-test to post-test and follow-up. This result suggests that individuals in a euthymic state at the baseline engaged more actively in the proposed group activities, which promote social interactions, thereby stimulating language skills. Severe depression has additive or multiplicative effects on cognitive deterioration (Piras et al., 2021) via a two-way process that underlies the interaction between cognition and mood. This is because some cognitive dysfunctions exacerbate an individual’s susceptibility to recurrent depression, while low mood drives deficits in cognitive functioning (Wiels et al., 2021). Our results are inconsistent with previous reports on the influence of individual characteristics on response to CST, however (Aguirre et al., 2013; Apoìstolo et al., 2014; Marinho et al., 2020). The discrepancies may relate to the different set of individual characteristics considered in the present study (vis-à-vis those explored by Aguirre et al., 2013; Apoìstolo et al., 2014), and to our having treated education as a continuous variable (whereas Marinho et al., 2020, split their sample into individuals with a high vs. low education).

A different picture emerged when secondary outcome measures were considered. Age, not education, revealed a role in predicting the short- and long-term benefits of CST, when mood and everyday functioning were explored at least. Age seemed to have a compensatory effect on the benefits of CST-IT on participants’ depressive symptoms. Older participants reported a reduction in their depressive symptoms from pre-test to post-test, which also seemed to persist in the longer term. Older age and the social stigma of dementia raise the risk of loneliness and isolation (Rewerska-Juśko and Rejdak, 2020), and consequent low mood (Holmén et al., 2000). The enriched environment and stimulating characteristics of CST—in terms of positive social interactions, engaging activities, and reinforced personhood—might compensate for the detrimental effect of older age on mood, improving the emotional-motivational attitude of older participants. Older age also seems to have a compensatory role in predicting everyday functioning: older participants were more likely to preserve their everyday functional ability (as measured by the DAD) than younger ones, whose functional dependence seemed to increase with time (at follow-up). This might be because the disease was progressing faster in the younger than in the older participants (Gardner et al., 2013), so the former were less able to profit from the proposed activities and “enriched” environment of CST programs. As for the QoL-AD, age and education showed a compensatory effect on the predicted effects of CST on quality of life, with older and less well-educated participants seeming to benefit slightly more. These results suggest that the activities involved are more likely to re-activate and support the residual skills and competences of the more severely impaired individuals: their lower baseline CR (education level) gives them a limited capacity to engage compensatory task-processing mechanisms, thereby prompting greater benefits in terms of quality of life, possibly mediated by positive changes in cognition (Woods et al., 2007).

It is also noteworthy that participants’ performance and scores in the outcome measures considered were also broadly affected by their baseline cognitive and/or mood and behavioral profiles, regardless of assessment session. A more or less impaired profile, particularly in terms of general cognitive functioning (i.e., higher MMSE scores), and the presence or absence of behavioral and neuropsychiatric symptoms (albeit very modestly and with a high degree of uncertainty) influenced performance in the ADAS-Cog and NLT (and QoL-AD scores). Intriguingly, more severe baseline depressive symptoms were associated with a better performance in the ADAS-Cog. One explanation for this finding might be that a worse performance in the ADAS-Cog, meaning a more severe cognitive impairment, is usually associated with an individual’s lesser awareness of their cognitive decline (Cacciamani et al., 2020), which might protect against affective symptoms like depression, anxiety, and apathy (Azocar et al., 2021). At the same time, a more impaired profile in terms of baseline behavioral and neuropsychiatric symptoms was associated with more severe depressive symptoms, and (albeit only modestly) with a worse everyday functioning. As for the NPI, apart from confirming the efficacy of CST-IT in counteracting the severity and frequency of behavioral and neuropsychiatric symptoms (see Carbone et al., 2021), after accounting for the influence of the individual characteristics considered, we found that NPI scores differed as a function of education in younger individuals with more severe baseline depressive symptoms. As well as confirming that education (as a proxy for CR) can mitigate the severity of behavioral and affective symptoms of dementia (Kwak et al., 2020), this pattern of results further corroborates the complex relationships between cognitive, depressive, behavioral and neuropsychiatric symptoms in people with mild-to-moderate dementia (Dillon et al., 2013), and the neurobiological link between mood disturbances, neural degeneration and cognitive impairment (e.g., Köhler et al., 2015; Andrews et al., 2018).

We need to draw attention to some limitations of our study, however. For the DAD and QoL-AD in particular, random effects (subjects and centers) explained a large part of the variance (compared with the other predictors), so any effects on these outcomes of the individual characteristics explored should be interpreted with caution. In addition, none of the individual characteristics considered here could explain the benefits of CST-IT once the severity and frequency of behavioral and neuropsychiatric symptoms (as measured with the NPI) had been taken into account.

Since the present study was exploratory in nature, the extent to which individual factors -other than those examined here- might affect the benefits of CST should be further explored, especially in crucial domains like functional ability and quality of life (see Lobbia et al., 2019; Marinho et al., 2020). We also did not consider other relevant clinical details relating to dementia (such as its early or late onset and duration, and the use of medication), which warrant further study. To what extent individuals with neurocognitive disorders prompted by different etiological mechanisms might benefit from CST is worth testing with an *ad hoc* sampling procedure because these patients’ cognitive, behavioral and neuropsychiatric symptoms evolve differently. This issue has not been addressed in CST studies (only one partially explored this issue; see Piras et al., 2017), but shedding light on it would enable the protocol to be fine adjusted to individual patients’ “residual resources” and “impairments,” in terms of their cognitive functioning, mood, behavioral and neuropsychiatric symptoms, and thereby maximize its efficacy. Finally, even using gold-standard tasks and questionnaires to assess the efficacy of interventions like CST (see Lobbia et al., 2019) seems unable to capture any changes induced by the intervention alone or its interaction with individual characteristics. Future studies should address this issue, and attempt to use more suitable measures in examining the interplay between individual characteristics (e.g., Borella et al., in preparation^3^) and specific symptoms (e.g., neuropsychiatric conditions like anxiety or apathy) likely to affect response to psychosocial interventions as CST.

Despite these limitations, our results suggest that the effects of individual characteristics on how PwD respond to CST might be hard to explain in the light of the compensation or magnification hypotheses alone (Lövdén et al., 2012). A more complex picture emerged, depending on the outcome measures considered, because some individual characteristics—particularly age and education (as a proxy of CR)—had both a magnifying effect on cognitive outcomes (education), and a compensatory effect on mood (age) after CST-IT. Such individual characteristics also made a modest compensatory contribution to the benefits of CST-IT on everyday functioning (age) and quality of life (age and education).

Overall, these findings are the first to underscore the importance of considering and further exploring the role of individual characteristics in accounting for any benefits of psychosocial interventions like CST, with a view to maximizing its efficacy for PwD with different socio-demographic and neuropathological profiles.

## Data Availability Statement

The data that support the findings of this study, the analytic methods, and study materials are available from the corresponding author upon reasonable request. Requests to access these datasets should be directed to EB, erika.borella@unipd.it.

## Ethics Statement

The studies involving human participants were reviewed and approved by the Ethical Committee for the Psychological Research, University of Padova, Italy. The patients/participants provided their written informed consent to participate in this study.

## Author Contributions

EC contributed to designing the study, analyzing and interpreting the data, and writing the manuscript. FP contributed to designing the study, interpreting the data, and writing the manuscript. MP contributed to analyzing and interpreting the data. EB designed the study and contributed to interpreting the data and writing the manuscript. All authors read and approved the final manuscript.

## Conflict of Interest

The authors declare that the research was conducted in the absence of any commercial or financial relationships that could be construed as a potential conflict of interest.

## Publisher’s Note

All claims expressed in this article are solely those of the authors and do not necessarily represent those of their affiliated organizations, or those of the publisher, the editors and the reviewers. Any product that may be evaluated in this article, or claim that may be made by its manufacturer, is not guaranteed or endorsed by the publisher.

## Footnotes

1 The protocol envisaged the inclusion of participants in CST program depending on their MMSE scores, but other factors should be considered as well, and as done in the present study. Patients scoring below 14 (but above 10) in the MMSE, who could properly understand, communicate, and manage the material proposed in the intervention/control conditions were eligible (this was true of 4.8% of the sample). Also, patients with MMSE scores over 24, but with other indicators (e.g., scores in other neuropsychological tests, demographics, clinical history) confirming a diagnosis of dementia could be considered eligible for CST (here they were 11% of the sample).

2 No parallel versions were used across the three assessment sessions due to the nature of the tests/inventories chosen as outcome measures (mainly classical neuropsychological tests and questionnaires known to be reliable in terms of their test-retest properties), and the features of participants’ neurocognitive disorders.

3 Borella, E., Ghisletta, P., Carbone, E., and Aichele, S. (in preparation). *The Current and Retrospective Cognitive Reserve (2CR) Survey.*
