# Supplementary material for: The Role of Individual Characteristics in Predicting Short- and Long-Term Cognitive and Psychological Benefits of Cognitive Stimulation Therapy for Mild-to-Moderate Dementia
Source: Front Aging Neurosci. 2022 Jan 11;13:811127. doi: 10.3389/fnagi.2021.811127 (PMC8787290; doi:10.3389/fnagi.2021.811127)
Supplement: Supplementary file 1 [file Data_Sheet_1.PDF]

Supplementary materials

The role of individual characteristics in predicting short- and long-term cognitive and psychological benefits of Cognitive Stimulation Therapy (CST) for mild-to-moderate dementia.

December 11, 2021

Contents

1 Descriptive statistics for the CST-IT group demographics, and for each measure of interest by assessment session, and description of the outcomes considered 2
1.1 Description of the outcomes . . . 3
1.1.1 Primary outcome measures . . . 3
1.1.2 Secondary outcome measures . . . 3

2 Models tested 4
2.1 Estimation details . . . 20
2.2 Priors choice . . . 20
2.3 Model comparison . . . 21

3 Results 22
3.1 General cognitive functioning and language . . . 23
3.1.1 Alzheimer's Disease Assessment Scale - Cognitive subscale (ADAS-Cog) . . . 23
3.1.2 Narrative Language Test (NLT) . . . 26
3.2 Mood and behavior . . . 30
3.2.1 Cornell Scale . . . 30
3.2.2 Neuropsychiatric Inventory (NPI) . . . 33
3.3 Everyday functioning and quality of life . . . 37
3.3.1 Disability Assessment for Dementia (DAD) . . . 37
3.3.2 Quality of Life- Alzheimer's Disease scale (QoL-AD) . . . 40

4 Appendix: Used R packages 43

References 44

1 Descriptive statistics for the CST-IT group demographics, and for each measure of interest by assessment session, and description of the outcomes considered

Table S1: Descriptive statistics for the CST-IT group demographics (69.1% female), and for each measure of interest at pre-test, post-test and follow-up.

	N	M	SD	Min-Max
Age	123	82.57	9.33	50-98
Education (years)	118	6.75	3.75	1-19
MMSE (baseline)	123	20.17	4.02	12-28

	Pre-test			Post-test			Follow-up		
	N	M	SD	N	M	SD	N	M	SD
ADAS-Cog	108	28.40	10.20	108	26.08	10.90	91	26.53	11.42
NLT	122	11.10	5.74	122	14.12	7.29	105	12.83	5.87
Cornell	123	6.07	5.63	123	4.16	4.38	106	5.04	4.99
NPI	123	11.77	13.41	123	8.93	10.91	106	13.44	15.70
DAD	84	54.26	24.04	84	53.50	24.74	69	53.59	22.83
QoL-AD	118	28.00	9.89	123	29.52	7.81	106	29.09	7.86

Notes: MMSE = Mini-Mental State Examination, ADAS-Cog = Alzheimer's Disease Assessment Scale - Cognitive subscale, NLT = Narrative Language Test, Cornell = Cornell test, NPI = Neuropsychiatric Inventory, DAD = Disability Assessment for Dementia, QoL-AD = Quality of Life - Alzheimer's Disease scale

1.1 Description of the outcomes

1.1.1 Primary outcome measures

General cognitive functioning – ADAS-Cog

Description: It contains 11 tasks assessing different cognitive domains (e.g., orientation, memory, language, praxis, attention).

Dependent variable: The total score (max = 70), with higher scores indicating a more impaired cognitive functioning.

Language – NLT

Description: Participants are asked to describe a single figure (the ‘Picnic‘ picture in the Western Aphasia Battery [Kertesz, 1982]), and then sets of figures (two cartoon sequences used by Nicholas & Brookshire, 1993). Descriptions are recorded, transcribed verbatim, and segmented using correct information unit analysis (Nicholas & Brookshire, 1993), followed by a quantitative textual analysis (Marini & Carlomagno, 2004).

Dependent variable: The sum of the correctly and accurately reported items.

1.1.2 Secondary outcome measures

Mood – Cornell scale (*)

Description: It contains 19 items assessing signs and symptoms of major depression in PwD. Each item is rated for severity on a scale from 0 (absent) to 2 (severe).

Dependent variable: The dependent variable was the sum of the scores for the 19 items. Scores were categorized as follow: 0-1 = no significant depressive symptoms, 1-10 = probable major depression, 10 and above = major depression.

Behavior – NPI (*)

Description: It assesses the frequency and severity of 12 behavioral and neuropsychiatric symptoms (e.g., delirium, hallucinations, apathy, anxiety, motor and sleep disturbances) in dementia patients.

Dependent variable: The dependent variable was the sum of the frequency x severity scores for each symptom, with higher scores indicating more frequent and more severe behavioral and neuropsychiatric disturbances.

Everyday functioning – DAD

Description: This covers basic, instrumental and leisure activities in 10 areas, from personal hygiene to managing money and medicines. The items in each area assess the individual’s ability in three dimensions: initiation (ability to decide and/or start an action); planning/organization (problem-solving and decision-making); and effective performance (ability to complete an action). The scores are: 1 (ability to perform the activity without help); 0 (inability to perform the activity); or N/A (activities never performed before the onset of the disease, or not performed in the past 2 weeks).

Dependent variable: The total score (ignoring items scored as N/A), obtained from the sum of all the scores, and setting the total number of valid answers in proportion to 100.

Quality of life – QoL-AD

Description: It includes 13 items assessing subjective components (e.g., perceived quality of life and psychological well-being) and objective components (e.g., behavioral competence and environment) of quality of life, rated by participants on a 4-point scale from 1 (poor) to 4 (excellent).

Dependent variable: The sum of all the items, where higher scores indicate a better quality of life.

Notes. ADAS-Cog: Alzheimer’s Disease Assessment Scale - Cognitive subscale; NLT: Narrative Language Test; DAD: Disability Assessment for Dementia; NPI: Neuropsychiatric Inventory; QoL-AD: Quality of Life - Alzheimer’s Disease scale. (*) The baseline (pre-test) scores of such outcome measures was used as predictor of CST benefits in the models.

2 Models tested

A mixed-effects approach was adopted for each measure of interest, i.e., the Alzheimer's Disease Assessment Scale - Cognitive subscale (ADAS-Cog), the Narrative Language Test, the Cornell scale and the Neuropsychiatric Inventory (NPI), the Disability Assessment for Dementia (DAD), and the Quality of Life - Alzheimer's Disease scale (QoL-AD). Assessment session (pre-test vs post-test vs follow-up), age, education and baseline (pre-test): i) Mini-Mental State Examination (MMSE), ii) Cornell scale (categorized as: 0-1= no depressive symptoms; 1-10 = subclinical depressive symptoms; 10-20 = depressive symptoms); and iii) NPI scores were added as predictors, and subjects and residential care homes (centers) as random effects.

The 375 models tested were the following[1]:

1. (Assessment session|subj) + (1|center)
2. Assessment session + (Assessment session|subj) + (1|center)
3. Age + Assessment session + (Assessment session|subj) + (1|center)
4. Age × Assessment session + (Assessment session|subj) + (1|center)
5. baseline Cornell + Assessment session + (Assessment session|subj) + (1|center)
6. baseline Cornell × Assessment session + (Assessment session|subj) + (1|center)
7. baseline MMSE + Assessment session + (Assessment session|subj) + (1|center)
8. baseline MMSE × Assessment session + (Assessment session|subj) + (1|center)
9. baseline NPI + Assessment session + (Assessment session|subj) + (1|center)
10. baseline NPI × Assessment session + (Assessment session|subj) + (1|center)
11. Education + Assessment session + (Assessment session|subj) + (1|center)
12. Education × Assessment session + (Assessment session|subj) + (1|center)
13. Age + baseline Cornell + Assessment session + (Assessment session|subj) + (1|center)
14. Age × baseline Cornell + Assessment session + (Assessment session|subj) + (1|center)
15. Age × Assessment session + baseline Cornell + (Assessment session|subj) + (1|center)
16. baseline Cornell × Assessment session + Age + (Assessment session|subj) + (1|center)
17. Age × baseline Cornell × Assessment session + (Assessment session|subj) + (1|center)
18. Age + baseline MMSE + Assessment session + (Assessment session|subj) + (1|center)
19. Age × baseline MMSE + Assessment session + (Assessment session|subj) + (1|center)
20. Age × Assessment session + baseline MMSE + (Assessment session|subj) + (1|center)
21. baseline MMSE × Assessment session + Age + (Assessment session|subj) + (1|center)
22. Age × baseline MMSE × Assessment session + (Assessment session|subj) + (1|center)
23. Age + baseline NPI + Assessment session + (Assessment session|subj) + (1|center)
24. Age × baseline NPI + Assessment session + (Assessment session|subj) + (1|center)
25. Age × Assessment session + baseline NPI + (Assessment session|subj) + (1|center)

[1]For the Cornell scale and the Neuropsychiatric Inventory (NPI), the models including the baseline (pre-test) performance in the Cornell scale and in the NPI as predictors, respectively, were not considered to avoid multicollinearity issues.

26. baseline NPI × Assessment session + Age + (Assessment session|subj) + (1|center)
27. Age × baseline NPI × Assessment session + (Assessment session|subj) + (1|center)
28. Age + Education + Assessment session + (Assessment session|subj) + (1|center)
29. Age × Education + Assessment session + (Assessment session|subj) + (1|center)
30. Age × Assessment session + Education + (Assessment session|subj) + (1|center)
31. Education × Assessment session + Age + (Assessment session|subj) + (1|center)
32. Age × Education × Assessment session + (Assessment session|subj) + (1|center)
33. baseline Cornell + baseline MMSE + Assessment session + (Assessment session|subj) + (1|center)
34. baseline Cornell × baseline MMSE + Assessment session + (Assessment session|subj) + (1|center)
35. baseline Cornell × Assessment session + baseline MMSE + (Assessment session|subj) + (1|center)
36. baseline MMSE × Assessment session + baseline Cornell + (Assessment session|subj) + (1|center)
37. baseline Cornell × baseline MMSE × Assessment session + (Assessment session|subj) + (1|center)
38. baseline Cornell + baseline NPI + Assessment session + (Assessment session|subj) + (1|center)
39. baseline Cornell × baseline NPI + Assessment session + (Assessment session|subj) + (1|center)
40. baseline Cornell × Assessment session + baseline NPI + (Assessment session|subj) + (1|center)
41. baseline NPI × Assessment session + baseline Cornell + (Assessment session|subj) + (1|center)
42. baseline Cornell × baseline NPI × Assessment session + (Assessment session|subj) + (1|center)
43. baseline Cornell + Education + Assessment session + (Assessment session|subj) + (1|center)
44. baseline Cornell × Education + Assessment session + (Assessment session|subj) + (1|center)
45. baseline Cornell × Assessment session + Education + (Assessment session|subj) + (1|center)
46. Education × Assessment session + baseline Cornell + (Assessment session|subj) + (1|center)
47. baseline Cornell × Education × Assessment session + (Assessment session|subj) + (1|center)
48. baseline MMSE + baseline NPI + Assessment session + (Assessment session|subj) + (1|center)
49. baseline MMSE × baseline NPI + Assessment session + (Assessment session|subj) + (1|center)
50. baseline MMSE × Assessment session + baseline NPI + (Assessment session|subj) + (1|center)
51. baseline NPI × Assessment session + baseline MMSE + (Assessment session|subj) + (1|center)
52. baseline MMSE × baseline NPI × Assessment session + (Assessment session|subj) + (1|center)
53. baseline MMSE + Education + Assessment session + (Assessment session|subj) + (1|center)
54. baseline MMSE × Education + Assessment session + (Assessment session|subj) + (1|center)
55. baseline MMSE × Assessment session + Education + (Assessment session|subj) + (1|center)
56. Education × Assessment session + baseline MMSE + (Assessment session|subj) + (1|center)
57. baseline MMSE × Education × Assessment session + (Assessment session|subj) + (1|center)
58. baseline NPI + Education + Assessment session + (Assessment session|subj) + (1|center)

59. baseline NPI × Education + Assessment session + (Assessment session|subj) + (1|center)
60. baseline NPI × Assessment session + Education + (Assessment session|subj) + (1|center)
61. Education × Assessment session + baseline NPI + (Assessment session|subj) + (1|center)
62. baseline NPI × Education × Assessment session + (Assessment session|subj) + (1|center)
63. Age + baseline Cornell + baseline MMSE + Assessment session + (Assessment session|subj) + (1|center)
64. Age × baseline Cornell + baseline MMSE + Assessment session + (Assessment session|subj) + (1|center)
65. Age × baseline MMSE + baseline Cornell + Assessment session + (Assessment session|subj) + (1|center)
66. Age × Assessment session + baseline Cornell + baseline MMSE + (Assessment session|subj) + (1|center)
67. baseline Cornell × baseline MMSE + Age + Assessment session + (Assessment session|subj) + (1|center)
68. baseline Cornell × Assessment session + Age + baseline MMSE + (Assessment session|subj) + (1|center)
69. baseline MMSE × Assessment session + Age + baseline Cornell + (Assessment session|subj) + (1|center)
70. Age × baseline Cornell × baseline MMSE + Assessment session + (Assessment session|subj) + (1|center)
71. Age × baseline Cornell × Assessment session + baseline MMSE + (Assessment session|subj) + (1|center)
72. Age × baseline MMSE × Assessment session + baseline Cornell + (Assessment session|subj) + (1|center)
73. baseline Cornell × baseline MMSE × Assessment session + Age + (Assessment session|subj) + (1|center)
74. Age × baseline Cornell × baseline MMSE × Assessment session + (Assessment session|subj) + (1|center)
75. Age + baseline Cornell + baseline NPI + Assessment session + (Assessment session|subj) + (1|center)
76. Age × baseline Cornell + baseline NPI + Assessment session + (Assessment session|subj) + (1|center)
77. Age × baseline NPI + baseline Cornell + Assessment session + (Assessment session|subj) + (1|center)
78. Age × Assessment session + baseline Cornell + baseline NPI + (Assessment session|subj) + (1|center)
79. baseline Cornell × baseline NPI + Age + Assessment session + (Assessment session|subj) + (1|center)
80. baseline Cornell × Assessment session + Age + baseline NPI + (Assessment session|subj) + (1|center)
81. baseline NPI × Assessment session + Age + baseline Cornell + (Assessment session|subj) + (1|center)
82. Age × baseline Cornell × baseline NPI + Assessment session + (Assessment session|subj) + (1|center)
83. Age × baseline Cornell × Assessment session + baseline NPI + (Assessment session|subj) + (1|center)
84. Age × baseline NPI × Assessment session + baseline Cornell + (Assessment session|subj) + (1|center)
85. baseline Cornell × baseline NPI × Assessment session + Age + (Assessment session|subj) + (1|center)
86. Age × baseline Cornell × baseline NPI × Assessment session + (Assessment session|subj) + (1|center)
87. Age + baseline Cornell + Education + Assessment session + (Assessment session|subj) + (1|center)
88. Age × baseline Cornell + Education + Assessment session + (Assessment session|subj) + (1|center)
89. Age × Education + baseline Cornell + Assessment session + (Assessment session|subj) + (1|center)
90. Age × Assessment session + baseline Cornell + Education + (Assessment session|subj) + (1|center)
91. baseline Cornell × Education + Age + Assessment session + (Assessment session|subj) + (1|center)

92. baseline Cornell × Assessment session + Age + Education + (Assessment session|subj) + (1|center)
93. Education × Assessment session + Age + baseline Cornell + (Assessment session|subj) + (1|center)
94. Age × baseline Cornell × Education + Assessment session + (Assessment session|subj) + (1|center)
95. Age × baseline Cornell × Assessment session + Education + (Assessment session|subj) + (1|center)
96. Age × Education × Assessment session + baseline Cornell + (Assessment session|subj) + (1|center)
97. baseline Cornell × Education × Assessment session + Age + (Assessment session|subj) + (1|center)
98. Age × baseline Cornell × Education × Assessment session + (Assessment session|subj) + (1|center)
99. Age + baseline MMSE + baseline NPI + Assessment session + (Assessment session|subj) + (1|center)
100. Age × baseline MMSE + baseline NPI + Assessment session + (Assessment session|subj) + (1|center)
101. Age × baseline NPI + baseline MMSE + Assessment session + (Assessment session|subj) + (1|center)
102. Age × Assessment session + baseline MMSE + baseline NPI + (Assessment session|subj) + (1|center)
103. baseline MMSE × baseline NPI + Age + Assessment session + (Assessment session|subj) + (1|center)
104. baseline MMSE × Assessment session + Age + baseline NPI + (Assessment session|subj) + (1|center)
105. baseline NPI × Assessment session + Age + baseline MMSE + (Assessment session|subj) + (1|center)
106. Age × baseline MMSE × baseline NPI + Assessment session + (Assessment session|subj) + (1|center)
107. Age × baseline MMSE × Assessment session + baseline NPI + (Assessment session|subj) + (1|center)
108. Age × baseline NPI × Assessment session + baseline MMSE + (Assessment session|subj) + (1|center)
109. baseline MMSE × baseline NPI × Assessment session + Age + (Assessment session|subj) + (1|center)
110. Age × baseline MMSE × baseline NPI × Assessment session + (Assessment session|subj) + (1|center)
111. Age + baseline MMSE + Education + Assessment session + (Assessment session|subj) + (1|center)
112. Age × baseline MMSE + Education + Assessment session + (Assessment session|subj) + (1|center)
113. Age × Education + baseline MMSE + Assessment session + (Assessment session|subj) + (1|center)
114. Age × Assessment session + baseline MMSE + Education + (Assessment session|subj) + (1|center)
115. baseline MMSE × Education + Age + Assessment session + (Assessment session|subj) + (1|center)
116. baseline MMSE × Assessment session + Age + Education + (Assessment session|subj) + (1|center)
117. Education × Assessment session + Age + baseline MMSE + (Assessment session|subj) + (1|center)
118. Age × baseline MMSE × Education + Assessment session + (Assessment session|subj) + (1|center)
119. Age × baseline MMSE × Assessment session + Education + (Assessment session|subj) + (1|center)
120. Age × Education × Assessment session + baseline MMSE + (Assessment session|subj) + (1|center)
121. baseline MMSE × Education × Assessment session + Age + (Assessment session|subj) + (1|center)
122. Age × baseline MMSE × Education × Assessment session + (Assessment session|subj) + (1|center)
123. Age + baseline NPI + Education + Assessment session + (Assessment session|subj) + (1|center)
124. Age × baseline NPI + Education + Assessment session + (Assessment session|subj) + (1|center)

125. Age × Education + baseline NPI + Assessment session + (Assessment session|subj) + (1|center)

126. Age × Assessment session + baseline NPI + Education + (Assessment session|subj) + (1|center)

127. baseline NPI × Education + Age + Assessment session + (Assessment session|subj) + (1|center)

128. baseline NPI × Assessment session + Age + Education + (Assessment session|subj) + (1|center)

129. Education × Assessment session + Age + baseline NPI + (Assessment session|subj) + (1|center)

130. Age × baseline NPI × Education + Assessment session + (Assessment session|subj) + (1|center)

131. Age × baseline NPI × Assessment session + Education + (Assessment session|subj) + (1|center)

132. Age × Education × Assessment session + baseline NPI + (Assessment session|subj) + (1|center)

133. baseline NPI × Education × Assessment session + Age + (Assessment session|subj) + (1|center)

134. Age × baseline NPI × Education × Assessment session + (Assessment session|subj) + (1|center)

135. baseline Cornell + baseline MMSE + baseline NPI + Assessment session + (Assessment session|subj) + (1|center)

136. baseline Cornell × baseline MMSE + baseline NPI + Assessment session + (Assessment session|subj) + (1|center)

137. baseline Cornell × baseline NPI + baseline MMSE + Assessment session + (Assessment session|subj) + (1|center)

138. baseline Cornell × Assessment session + baseline MMSE + baseline NPI + (Assessment session|subj) + (1|center)

139. baseline MMSE × baseline NPI + baseline Cornell + Assessment session + (Assessment session|subj) + (1|center)

140. baseline MMSE × Assessment session + baseline Cornell + baseline NPI + (Assessment session|subj) + (1|center)

141. baseline NPI × Assessment session + baseline Cornell + baseline MMSE + (Assessment session|subj) + (1|center)

142. baseline Cornell × baseline MMSE × baseline NPI + Assessment session + (Assessment session|subj) + (1|center)

143. baseline Cornell × baseline MMSE × Assessment session + baseline NPI + (Assessment session|subj) + (1|center)

144. baseline Cornell × baseline NPI × Assessment session + baseline MMSE + (Assessment session|subj) + (1|center)

145. baseline MMSE × baseline NPI × Assessment session + baseline Cornell + (Assessment session|subj) + (1|center)

146. baseline Cornell × baseline MMSE × baseline NPI × Assessment session + (Assessment session|subj) + (1|center)

147. baseline Cornell + baseline MMSE + Education + Assessment session + (Assessment session|subj) + (1|center)

148. baseline Cornell × baseline MMSE + Education + Assessment session + (Assessment session|subj) + (1|center)

149. baseline Cornell × Education + baseline MMSE + Assessment session + (Assessment session|subj) + (1|center)

150. baseline Cornell × Assessment session + baseline MMSE + Education + (Assessment session|subj) + (1|center)

151. baseline MMSE × Education + baseline Cornell + Assessment session + (Assessment session|subj) + (1|center)

152. baseline MMSE × Assessment session + baseline Cornell + Education + (Assessment session|subj) + (1|center)

153. Education × Assessment session + baseline Cornell + baseline MMSE + (Assessment session|subj) + (1|center)

154. baseline Cornell × baseline MMSE × Education + Assessment session + (Assessment session|subj) + (1|center)

155. baseline Cornell × baseline MMSE × Assessment session + Education + (Assessment session|subj) + (1|center)

156. baseline Cornell × Education × Assessment session + baseline MMSE + (Assessment session|subj) + (1|center)

157. baseline MMSE × Education × Assessment session + baseline Cornell + (Assessment session|subj) + (1|center)

158. baseline Cornell × baseline MMSE × Education × Assessment session + (Assessment session|subj) + (1|center)

159. baseline Cornell + baseline NPI + Education + Assessment session + (Assessment session|subj) + (1|center)

160. baseline Cornell × baseline NPI + Education + Assessment session + (Assessment session|subj) + (1|center)

161. baseline Cornell × Education + baseline NPI + Assessment session + (Assessment session|subj) + (1|center)

162. baseline Cornell × Assessment session + baseline NPI + Education + (Assessment session|subj) + (1|center)

163. baseline NPI × Education + baseline Cornell + Assessment session + (Assessment session|subj) + (1|center)

164. baseline NPI × Assessment session + baseline Cornell + Education + (Assessment session|subj) + (1|center)

165. Education × Assessment session + baseline Cornell + baseline NPI + (Assessment session|subj) + (1|center)

166. baseline Cornell × baseline NPI × Education + Assessment session + (Assessment session|subj) + (1|center)

167. baseline Cornell × baseline NPI × Assessment session + Education + (Assessment session|subj) + (1|center)

168. baseline Cornell × Education × Assessment session + baseline NPI + (Assessment session|subj) + (1|center)

169. baseline NPI × Education × Assessment session + baseline Cornell + (Assessment session|subj) + (1|center)

170. baseline Cornell × baseline NPI × Education × Assessment session + (Assessment session|subj) + (1|center)

171. baseline MMSE + baseline NPI + Education + Assessment session + (Assessment session|subj) + (1|center)

172. baseline MMSE × baseline NPI + Education + Assessment session + (Assessment session|subj) + (1|center)

173. baseline MMSE × Education + baseline NPI + Assessment session + (Assessment session|subj) + (1|center)

174. baseline MMSE × Assessment session + baseline NPI + Education + (Assessment session|subj) + (1|center)

175. baseline NPI × Education + baseline MMSE + Assessment session + (Assessment session|subj) + (1|center)

176. baseline NPI × Assessment session + baseline MMSE + Education + (Assessment session|subj) + (1|center)

177. Education × Assessment session + baseline MMSE + baseline NPI + (Assessment session|subj) + (1|center)

178. baseline MMSE × baseline NPI × Education + Assessment session + (Assessment session|subj) + (1|center)

179. baseline MMSE × baseline NPI × Assessment session + Education + (Assessment session|subj) + (1|center)

180. baseline MMSE × Education × Assessment session + baseline NPI + (Assessment session|subj) + (1|center)

181. baseline NPI × Education × Assessment session + baseline MMSE + (Assessment session|subj) + (1|center)

182. baseline MMSE × baseline NPI × Education × Assessment session + (Assessment session|subj) + (1|center)

183. Age + baseline Cornell + baseline MMSE + baseline NPI + Assessment session + (Assessment session|subj) + (1|center)

184. Age × baseline Cornell + baseline MMSE + baseline NPI + Assessment session + (Assessment session|subj) + (1|center)

185. Age × baseline MMSE + baseline Cornell + baseline NPI + Assessment session + (Assessment session|subj) + (1|center)

186. Age × baseline NPI + baseline Cornell + baseline MMSE + Assessment session + (Assessment session|subj) + (1|center)

187. Age × Assessment session + baseline Cornell + baseline MMSE + baseline NPI + (Assessment session|subj) + (1|center)

188. baseline Cornell × baseline MMSE + Age + baseline NPI + Assessment session + (Assessment session|subj) + (1|center)

189. baseline Cornell × baseline NPI + Age + baseline MMSE + Assessment session + (Assessment session|subj) + (1|center)

190. baseline Cornell × Assessment session + Age + baseline MMSE + baseline NPI + (Assessment session|subj) + (1|center)

191. baseline MMSE × baseline NPI + Age + baseline Cornell + Assessment session + (Assessment session|subj) + (1|center)
192. baseline MMSE × Assessment session + Age + baseline Cornell + baseline NPI + (Assessment session|subj) + (1|center)
193. baseline NPI × Assessment session + Age + baseline Cornell + baseline MMSE + (Assessment session|subj) + (1|center)
194. Age × baseline Cornell × baseline MMSE + baseline NPI + Assessment session + (Assessment session|subj) + (1|center)
195. Age × baseline Cornell × baseline NPI + baseline MMSE + Assessment session + (Assessment session|subj) + (1|center)
196. Age × baseline Cornell × Assessment session + baseline MMSE + baseline NPI + (Assessment session|subj) + (1|center)
197. Age × baseline MMSE × baseline NPI + baseline Cornell + Assessment session + (Assessment session|subj) + (1|center)
198. Age × baseline MMSE × Assessment session + baseline Cornell + baseline NPI + (Assessment session|subj) + (1|center)
199. Age × baseline NPI × Assessment session + baseline Cornell + baseline MMSE + (Assessment session|subj) + (1|center)
200. baseline Cornell × baseline MMSE × baseline NPI + Age + Assessment session + (Assessment session|subj) + (1|center)
201. baseline Cornell × baseline MMSE × Assessment session + Age + baseline NPI + (Assessment session|subj) + (1|center)
202. baseline Cornell × baseline NPI × Assessment session + Age + baseline MMSE + (Assessment session|subj) + (1|center)
203. baseline MMSE × baseline NPI × Assessment session + Age + baseline Cornell + (Assessment session|subj) + (1|center)
204. Age × baseline Cornell × baseline MMSE × baseline NPI + Assessment session + (Assessment session|subj) + (1|center)
205. Age × baseline Cornell × baseline MMSE × Assessment session + baseline NPI + (Assessment session|subj) + (1|center)
206. Age × baseline Cornell × baseline NPI × Assessment session + baseline MMSE + (Assessment session|subj) + (1|center)
207. Age × baseline MMSE × baseline NPI × Assessment session + baseline Cornell + (Assessment session|subj) + (1|center)
208. baseline Cornell × baseline MMSE × baseline NPI × Assessment session + Age + (Assessment session|subj) + (1|center)
209. Age × baseline Cornell × baseline MMSE × baseline NPI × Assessment session + (Assessment session|subj) + (1|center)
210. Age + baseline Cornell + baseline MMSE + Education + Assessment session + (Assessment session|subj) + (1|center)
211. Age × baseline Cornell + baseline MMSE + Education + Assessment session + (Assessment session|subj) + (1|center)

212. Age × baseline MMSE + baseline Cornell + Education + Assessment session + (Assessment session|subj) + (1|center)

213. Age × Education + baseline Cornell + baseline MMSE + Assessment session + (Assessment session|subj) + (1|center)

214. Age × Assessment session + baseline Cornell + baseline MMSE + Education + (Assessment session|subj) + (1|center)

215. baseline Cornell × baseline MMSE + Age + Education + Assessment session + (Assessment session|subj) + (1|center)

216. baseline Cornell × Education + Age + baseline MMSE + Assessment session + (Assessment session|subj) + (1|center)

217. baseline Cornell × Assessment session + Age + baseline MMSE + Education + (Assessment session|subj) + (1|center)

218. baseline MMSE × Education + Age + baseline Cornell + Assessment session + (Assessment session|subj) + (1|center)

219. baseline MMSE × Assessment session + Age + baseline Cornell + Education + (Assessment session|subj) + (1|center)

220. Education × Assessment session + Age + baseline Cornell + baseline MMSE + (Assessment session|subj) + (1|center)

221. Age × baseline Cornell × baseline MMSE + Education + Assessment session + (Assessment session|subj) + (1|center)

222. Age × baseline Cornell × Education + baseline MMSE + Assessment session + (Assessment session|subj) + (1|center)

223. Age × baseline Cornell × Assessment session + baseline MMSE + Education + (Assessment session|subj) + (1|center)

224. Age × baseline MMSE × Education + baseline Cornell + Assessment session + (Assessment session|subj) + (1|center)

225. Age × baseline MMSE × Assessment session + baseline Cornell + Education + (Assessment session|subj) + (1|center)

226. Age × Education × Assessment session + baseline Cornell + baseline MMSE + (Assessment session|subj) + (1|center)

227. baseline Cornell × baseline MMSE × Education + Age + Assessment session + (Assessment session|subj) + (1|center)

228. baseline Cornell × baseline MMSE × Assessment session + Age + Education + (Assessment session|subj) + (1|center)

229. baseline Cornell × Education × Assessment session + Age + baseline MMSE + (Assessment session|subj) + (1|center)

230. baseline MMSE × Education × Assessment session + Age + baseline Cornell + (Assessment session|subj) + (1|center)

231. Age × baseline Cornell × baseline MMSE × Education + Assessment session + (Assessment session|subj) + (1|center)

232. Age × baseline Cornell × baseline MMSE × Assessment session + Education + (Assessment session|subj) + (1|center)

233. Age × baseline Cornell × Education × Assessment session + baseline MMSE + (Assessment session|subj) + (1|center)

234. Age × baseline MMSE × Education × Assessment session + baseline Cornell + (Assessment session|subj) + (1|center)

235. baseline Cornell × baseline MMSE × Education × Assessment session + Age + (Assessment session|subj) + (1|center)

236. Age × baseline Cornell × baseline MMSE × Education × Assessment session + (Assessment session|subj) + (1|center)

237. Age + baseline Cornell + baseline NPI + Education + Assessment session + (Assessment session|subj) + (1|center)

238. Age × baseline Cornell + baseline NPI + Education + Assessment session + (Assessment session|subj) + (1|center)

239. Age × baseline NPI + baseline Cornell + Education + Assessment session + (Assessment session|subj) + (1|center)

240. Age × Education + baseline Cornell + baseline NPI + Assessment session + (Assessment session|subj) + (1|center)

241. Age × Assessment session + baseline Cornell + baseline NPI + Education + (Assessment session|subj) + (1|center)

242. baseline Cornell × baseline NPI + Age + Education + Assessment session + (Assessment session|subj) + (1|center)

243. baseline Cornell × Education + Age + baseline NPI + Assessment session + (Assessment session|subj) + (1|center)

244. baseline Cornell × Assessment session + Age + baseline NPI + Education + (Assessment session|subj) + (1|center)

245. baseline NPI × Education + Age + baseline Cornell + Assessment session + (Assessment session|subj) + (1|center)

246. baseline NPI × Assessment session + Age + baseline Cornell + Education + (Assessment session|subj) + (1|center)

247. Education × Assessment session + Age + baseline Cornell + baseline NPI + (Assessment session|subj) + (1|center)

248. Age × baseline Cornell × baseline NPI + Education + Assessment session + (Assessment session|subj) + (1|center)

249. Age × baseline Cornell × Education + baseline NPI + Assessment session + (Assessment session|subj) + (1|center)

250. Age × baseline Cornell × Assessment session + baseline NPI + Education + (Assessment session|subj) + (1|center)

251. Age × baseline NPI × Education + baseline Cornell + Assessment session + (Assessment session|subj) + (1|center)

252. Age × baseline NPI × Assessment session + baseline Cornell + Education + (Assessment session|subj) + (1|center)

253. Age × Education × Assessment session + baseline Cornell + baseline NPI + (Assessment session|subj) + (1|center)

254. baseline Cornell × baseline NPI × Education + Age + Assessment session + (Assessment session|subj) + (1|center)

255. baseline Cornell × baseline NPI × Assessment session + Age + Education + (Assessment session|subj) + (1|center)

256. baseline Cornell × Education × Assessment session + Age + baseline NPI + (Assessment session|subj) + (1|center)

257. baseline NPI × Education × Assessment session + Age + baseline Cornell + (Assessment session|subj) + (1|center)

258. Age × baseline Cornell × baseline NPI × Education + Assessment session + (Assessment session|subj) + (1|center)

259. Age × baseline Cornell × baseline NPI × Assessment session + Education + (Assessment session|subj) + (1|center)

260. Age × baseline Cornell × Education × Assessment session + baseline NPI + (Assessment session|subj) + (1|center)

261. Age × baseline NPI × Education × Assessment session + baseline Cornell + (Assessment session|subj) + (1|center)

262. baseline Cornell × baseline NPI × Education × Assessment session + Age + (Assessment session|subj) + (1|center)

263. Age × baseline Cornell × baseline NPI × Education × Assessment session + (Assessment session|subj) + (1|center)

264. Age + baseline MMSE + baseline NPI + Education + Assessment session + (Assessment session|subj) + (1|center)

265. Age × baseline MMSE + baseline NPI + Education + Assessment session + (Assessment session|subj) + (1|center)

266. Age × baseline NPI + baseline MMSE + Education + Assessment session + (Assessment session|subj) + (1|center)

267. Age × Education + baseline MMSE + baseline NPI + Assessment session + (Assessment session|subj) + (1|center)

268. Age × Assessment session + baseline MMSE + baseline NPI + Education + (Assessment session|subj) + (1|center)

269. baseline MMSE × baseline NPI + Age + Education + Assessment session + (Assessment session|subj) + (1|center)

270. baseline MMSE × Education + Age + baseline NPI + Assessment session + (Assessment session|subj) + (1|center)

271. baseline MMSE × Assessment session + Age + baseline NPI + Education + (Assessment session|subj) + (1|center)

272. baseline NPI × Education + Age + baseline MMSE + Assessment session + (Assessment session|subj) + (1|center)

273. baseline NPI × Assessment session + Age + baseline MMSE + Education + (Assessment session|subj) + (1|center)

274. Education × Assessment session + Age + baseline MMSE + baseline NPI + (Assessment session|subj) + (1|center)

275. Age × baseline MMSE × baseline NPI + Education + Assessment session + (Assessment session|subj) + (1|center)

276. Age × baseline MMSE × Education + baseline NPI + Assessment session + (Assessment session|subj) + (1|center)

277. Age × baseline MMSE × Assessment session + baseline NPI + Education + (Assessment session|subj) + (1|center)

278. Age × baseline NPI × Education + baseline MMSE + Assessment session + (Assessment session|subj) + (1|center)

279. Age × baseline NPI × Assessment session + baseline MMSE + Education + (Assessment session|subj) + (1|center)

280. Age × Education × Assessment session + baseline MMSE + baseline NPI + (Assessment session|subj) + (1|center)

281. baseline MMSE × baseline NPI × Education + Age + Assessment session + (Assessment session|subj) + (1|center)

282. baseline MMSE × baseline NPI × Assessment session + Age + Education + (Assessment session|subj) + (1|center)

283. baseline MMSE × Education × Assessment session + Age + baseline NPI + (Assessment session|subj) + (1|center)

284. baseline NPI × Education × Assessment session + Age + baseline MMSE + (Assessment session|subj) + (1|center)

285. Age × baseline MMSE × baseline NPI × Education + Assessment session + (Assessment session|subj) + (1|center)

286. Age × baseline MMSE × baseline NPI × Assessment session + Education + (Assessment session|subj) + (1|center)

287. Age × baseline MMSE × Education × Assessment session + baseline NPI + (Assessment session|subj) + (1|center)

288. Age × baseline NPI × Education × Assessment session + baseline MMSE + (Assessment session|subj) + (1|center)

289. baseline MMSE × baseline NPI × Education × Assessment session + Age + (Assessment session|subj) + (1|center)

290. Age × baseline MMSE × baseline NPI × Education × Assessment session + (Assessment session|subj) + (1|center)

291. baseline Cornell + baseline MMSE + baseline NPI + Education + Assessment session + (Assessment session|subj) + (1|center)

292. baseline Cornell × baseline MMSE + baseline NPI + Education + Assessment session + (Assessment session|subj) + (1|center)

293. baseline Cornell × baseline NPI + baseline MMSE + Education + Assessment session + (Assessment session|subj) + (1|center)

294. baseline Cornell × Education + baseline MMSE + baseline NPI + Assessment session + (Assessment session|subj) + (1|center)

295. baseline Cornell × Assessment session + baseline MMSE + baseline NPI + Education + (Assessment session|subj) + (1|center)

296. baseline MMSE × baseline NPI + baseline Cornell + Education + Assessment session + (Assessment session|subj) + (1|center)

297. baseline MMSE × Education + baseline Cornell + baseline NPI + Assessment session + (Assessment session|subj) + (1|center)

298. baseline MMSE × Assessment session + baseline Cornell + baseline NPI + Education + (Assessment session|subj) + (1|center)

299. baseline NPI × Education + baseline Cornell + baseline MMSE + Assessment session + (Assessment session|subj) + (1|center)

300. baseline NPI × Assessment session + baseline Cornell + baseline MMSE + Education + (Assessment session|subj) + (1|center)

301. Education × Assessment session + baseline Cornell + baseline MMSE + baseline NPI + (Assessment session|subj) + (1|center)

302. baseline Cornell × baseline MMSE × baseline NPI + Education + Assessment session + (Assessment session|subj) + (1|center)

303. baseline Cornell × baseline MMSE × Education + baseline NPI + Assessment session + (Assessment session|subj) + (1|center)

304. baseline Cornell × baseline MMSE × Assessment session + baseline NPI + Education + (Assessment session|subj) + (1|center)

305. baseline Cornell × baseline NPI × Education + baseline MMSE + Assessment session + (Assessment session|subj) + (1|center)

306. baseline Cornell × baseline NPI × Assessment session + baseline MMSE + Education + (Assessment session|subj) + (1|center)

307. baseline Cornell × Education × Assessment session + baseline MMSE + baseline NPI + (Assessment session|subj) + (1|center)

308. baseline MMSE × baseline NPI × Education + baseline Cornell + Assessment session + (Assessment session|subj) + (1|center)

309. baseline MMSE × baseline NPI × Assessment session + baseline Cornell + Education + (Assessment session|subj) + (1|center)

310. baseline MMSE × Education × Assessment session + baseline Cornell + baseline NPI + (Assessment session|subj) + (1|center)

311. baseline NPI × Education × Assessment session + baseline Cornell + baseline MMSE + (Assessment session|subj) + (1|center)

312. baseline Cornell × baseline MMSE × baseline NPI × Education + Assessment session + (Assessment session|subj) + (1|center)

313. baseline Cornell × baseline MMSE × baseline NPI × Assessment session + Education + (Assessment session|subj) + (1|center)

314. baseline Cornell × baseline MMSE × Education × Assessment session + baseline NPI + (Assessment session|subj) + (1|center)

315. baseline Cornell × baseline NPI × Education × Assessment session + baseline MMSE + (Assessment session|subj) + (1|center)

316. baseline MMSE × baseline NPI × Education × Assessment session + baseline Cornell + (Assessment session|subj) + (1|center)

317. baseline Cornell × baseline MMSE × baseline NPI × Education × Assessment session + (Assessment session|subj) + (1|center)

318. Age + baseline Cornell + baseline MMSE + baseline NPI + Education + Assessment session + (Assessment session|subj) + (1|center)

319. Age × baseline Cornell + baseline MMSE + baseline NPI + Education + Assessment session + (Assessment session|subj) + (1|center)

320. Age × baseline MMSE + baseline Cornell + baseline NPI + Education + Assessment session + (Assessment session|subj) + (1|center)

321. Age × baseline NPI + baseline Cornell + baseline MMSE + Education + Assessment session + (Assessment session|subj) + (1|center)

322. Age × Education + baseline Cornell + baseline MMSE + baseline NPI + Assessment session + (Assessment session|subj) + (1|center)

323. Age × Assessment session + baseline Cornell + baseline MMSE + baseline NPI + Education + (Assessment session|subj) + (1|center)

324. baseline Cornell × baseline MMSE + Age + baseline NPI + Education + Assessment session + (Assessment session|subj) + (1|center)

325. baseline Cornell × baseline NPI + Age + baseline MMSE + Education + Assessment session + (Assessment session|subj) + (1|center)

326. baseline Cornell × Education + Age + baseline MMSE + baseline NPI + Assessment session + (Assessment session|subj) + (1|center)

327. baseline Cornell × Assessment session + Age + baseline MMSE + baseline NPI + Education + (Assessment session|subj) + (1|center)

328. baseline MMSE × baseline NPI + Age + baseline Cornell + Education + Assessment session + (Assessment session|subj) + (1|center)

329. baseline MMSE × Education + Age + baseline Cornell + baseline NPI + Assessment session + (Assessment session|subj) + (1|center)

330. baseline MMSE × Assessment session + Age + baseline Cornell + baseline NPI + Education + (Assessment session|subj) + (1|center)

331. baseline NPI × Education + Age + baseline Cornell + baseline MMSE + Assessment session + (Assessment session|subj) + (1|center)

332. baseline NPI × Assessment session + Age + baseline Cornell + baseline MMSE + Education + (Assessment session|subj) + (1|center)

333. Education × Assessment session + Age + baseline Cornell + baseline MMSE + baseline NPI + (Assessment session|subj) + (1|center)

334. Age × baseline Cornell × baseline MMSE + baseline NPI + Education + Assessment session + (Assessment session|subj) + (1|center)

335. Age × baseline Cornell × baseline NPI + baseline MMSE + Education + Assessment session + (Assessment session|subj) + (1|center)

336. Age × baseline Cornell × Education + baseline MMSE + baseline NPI + Assessment session + (Assessment session|subj) + (1|center)

337. Age × baseline Cornell × Assessment session + baseline MMSE + baseline NPI + Education + (Assessment session|subj) + (1|center)

338. Age × baseline MMSE × baseline NPI + baseline Cornell + Education + Assessment session + (Assessment session|subj) + (1|center)
339. Age × baseline MMSE × Education + baseline Cornell + baseline NPI + Assessment session + (Assessment session|subj) + (1|center)
340. Age × baseline MMSE × Assessment session + baseline Cornell + baseline NPI + Education + (Assessment session|subj) + (1|center)
341. Age × baseline NPI × Education + baseline Cornell + baseline MMSE + Assessment session + (Assessment session|subj) + (1|center)
342. Age × baseline NPI × Assessment session + baseline Cornell + baseline MMSE + Education + (Assessment session|subj) + (1|center)
343. Age × Education × Assessment session + baseline Cornell + baseline MMSE + baseline NPI + (Assessment session|subj) + (1|center)
344. baseline Cornell × baseline MMSE × baseline NPI + Age + Education + Assessment session + (Assessment session|subj) + (1|center)
345. baseline Cornell × baseline MMSE × Education + Age + baseline NPI + Assessment session + (Assessment session|subj) + (1|center)
346. baseline Cornell × baseline MMSE × Assessment session + Age + baseline NPI + Education + (Assessment session|subj) + (1|center)
347. baseline Cornell × baseline NPI × Education + Age + baseline MMSE + Assessment session + (Assessment session|subj) + (1|center)
348. baseline Cornell × baseline NPI × Assessment session + Age + baseline MMSE + Education + (Assessment session|subj) + (1|center)
349. baseline Cornell × Education × Assessment session + Age + baseline MMSE + baseline NPI + (Assessment session|subj) + (1|center)
350. baseline MMSE × baseline NPI × Education + Age + baseline Cornell + Assessment session + (Assessment session|subj) + (1|center)
351. baseline MMSE × baseline NPI × Assessment session + Age + baseline Cornell + Education + (Assessment session|subj) + (1|center)
352. baseline MMSE × Education × Assessment session + Age + baseline Cornell + baseline NPI + (Assessment session|subj) + (1|center)
353. baseline NPI × Education × Assessment session + Age + baseline Cornell + baseline MMSE + (Assessment session|subj) + (1|center)
354. Age × baseline Cornell × baseline MMSE × baseline NPI + Education + Assessment session + (Assessment session|subj) + (1|center)
355. Age × baseline Cornell × baseline MMSE × Education + baseline NPI + Assessment session + (Assessment session|subj) + (1|center)
356. Age × baseline Cornell × baseline MMSE × Assessment session + baseline NPI + Education + (Assessment session|subj) + (1|center)
357. Age × baseline Cornell × baseline NPI × Education + baseline MMSE + Assessment session + (Assessment session|subj) + (1|center)
358. Age × baseline Cornell × baseline NPI × Assessment session + baseline MMSE + Education + (Assessment session|subj) + (1|center)

359. Age × baseline Cornell × Education × Assessment session + baseline MMSE + baseline NPI + (Assessment session|subj) + (1|center)

360. Age × baseline MMSE × baseline NPI × Education + baseline Cornell + Assessment session + (Assessment session|subj) + (1|center)

361. Age × baseline MMSE × baseline NPI × Assessment session + baseline Cornell + Education + (Assessment session|subj) + (1|center)

362. Age × baseline MMSE × Education × Assessment session + baseline Cornell + baseline NPI + (Assessment session|subj) + (1|center)

363. Age × baseline NPI × Education × Assessment session + baseline Cornell + baseline MMSE + (Assessment session|subj) + (1|center)

364. baseline Cornell × baseline MMSE × baseline NPI × Education + Age + Assessment session + (Assessment session|subj) + (1|center)

365. baseline Cornell × baseline MMSE × baseline NPI × Assessment session + Age + Education + (Assessment session|subj) + (1|center)

366. baseline Cornell × baseline MMSE × Education × Assessment session + Age + baseline NPI + (Assessment session|subj) + (1|center)

367. baseline Cornell × baseline NPI × Education × Assessment session + Age + baseline MMSE + (Assessment session|subj) + (1|center)

368. baseline MMSE × baseline NPI × Education × Assessment session + Age + baseline Cornell + (Assessment session|subj) + (1|center)

369. Age × baseline Cornell × baseline MMSE × baseline NPI × Education + Assessment session + (Assessment session|subj) + (1|center)

370. Age × baseline Cornell × baseline MMSE × baseline NPI × Assessment session + Education + (Assessment session|subj) + (1|center)

371. Age × baseline Cornell × baseline MMSE × Education × Assessment session + baseline NPI + (Assessment session|subj) + (1|center)

372. Age × baseline Cornell × baseline NPI × Education × Assessment session + baseline MMSE + (Assessment session|subj) + (1|center)

373. Age × baseline MMSE × baseline NPI × Education × Assessment session + baseline Cornell + (Assessment session|subj) + (1|center)

374. baseline Cornell × baseline MMSE × baseline NPI × Education × Assessment session + Age + (Assessment session|subj) + (1|center)

375. Age × baseline Cornell × baseline MMSE × baseline NPI × Education × Assessment session + (Assessment session|subj) + (1|center)

The terms (Assessment session|subject) and (1|center) indicate the random effects.

2.1 Estimation details

The parameters of the models were fitted using a full Bayesian approach. The `brms` R-package (Bürkner, 2017, 2018) was used, which adopts a MCMC (Markov Chain Monte Carlo) estimation method implemented via STAN (Stan Development Team, 2019). Posterior distributions for each parameter were estimated using 4 MCMC chains, each running at least 4000 replications. The actual number of posterior samples for each parameter was 8000. MCMC convergence was assessed by using two indicators: i) Rhat (*potential scale reduction factor*; Gelman & Rubin, 1992), which assesses the level of convergence of the MCMC chains – values close to 1 indicate that chains converged –; and ii) Bulk ESS (Vehtari, Gelman, Simpson, Carpenter, & Bürkner, 2021), which assesses the effective sample size, i.e., it estimates the number of samples at posterior that are actually independent – it should generally be higher, at least 100 times higher than the number of chains, therefore $100 \times 4 = 400$ in our case –.

2.2 Priors choice

Weak informative priors were used for the regression coefficients (see Fig. S1). As for the Assessment session and interaction parameters, a Student's t (3,0,1) was used, i.e., priors assumed the values of such parameters to fall into the [-1,1] interval with a 48% probability. Concerning Age and baseline MMSE, a Student's t (3,0,.5) was used, i.e., priors assumed the values of such parameters to display a positive association with the outcome measures (the more aged and cognitively preserved participants benefit most) with a 93% probability. As for Education and baseline NPI, a Student's t (3,-1,.5) was used, i.e., priors assumed the values of such parameters to display a negative association with the outcome measures (the more educated and compromised -in terms of psychological and behavioral symptoms- participants benefit the less) with a 93% probability. Finally, for baseline Cornell scores, a Student's t (3,-.7, .5) and a Student's t (3,-1.4,.5) were used for the 1-10 = subclinical depressive symptoms and the 10-20 = depressive symptoms sub-categories respectively. That is, priors assumed that participants with more severe depressive symptoms benefit less than those showing a subclinical symptomatology or no depressive symptoms.

Default priors were settled, instead, for intercepts (Student's t zero centered distributions and with a

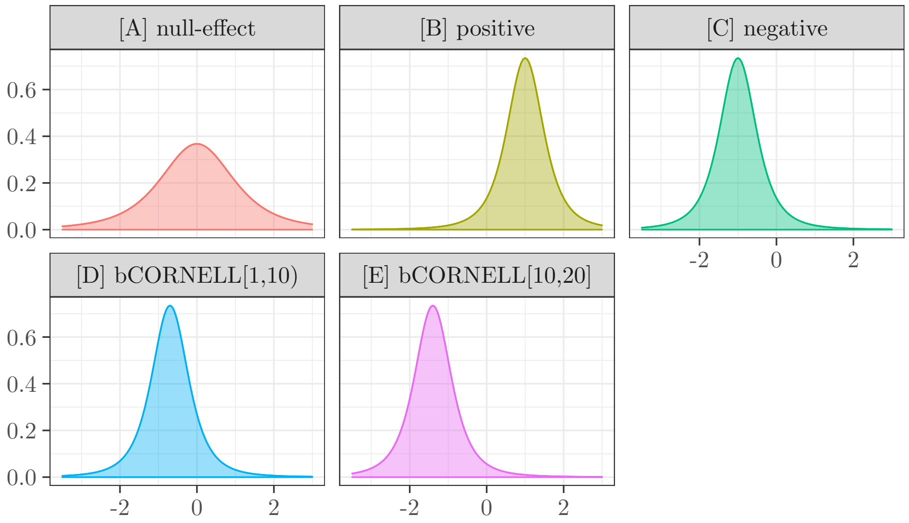

Figure S1: Weak informative priors. Each panel represents the prior used for each specific regression coefficient. [A] skeptical prior, used for the Assessment session coefficients and interactions parameters; [B] weak informative prior, used for parameters expressing positive associations (i.e., Age, baseline MMSE); [C] weak informative prior, used for parameters expressing negative associations (i.e., Education, baseline NPI); [D] and [E] weak informative priors, for baseline Cornell scores related to 1-10 = subclinical depressive symptoms and 10-20 = depressive symptoms sub-categories, respectively.

variability proportional to the one of the outcome measures considered), standard deviations of the random effects and the residuals (truncated Student's t distributions with a variability proportional to the one of the outcome measures considered) and LKJ distribution(Lewandowski, Kurowicka, & Joe, 2009) for correlations parameters.

2.3 Model comparison

A model-comparison strategy was used to identify the best model for each outcome measure of interest, based on the following performance indices: the Leave-One-Out cross-validation Information Criterion (LOO; Vehtari, Gelman, & Gabry, 2017) the Bayesian R^2 (Gelman, Goodrich, Gabry, & Vehtari, 2019), and the model weights (w; Yao, Vehtari, Simpson, & Gelman, 2018). Lower values of LOO, and higher values of w indicate a more plausible model. Given that the number of models to be tested made it impossible to compare them all simultaneously, for each of the outcome measure the parameters of each of the model were estimated, and for those models reaching convergence the LOO was calculated. Then, among the models reaching convergence, the 10 showing the lower LOO were compared with the stacking method (Yao et al., 2018) in order to compute the corresponding 10 values of w.

3 Results

For each measure of interest, the following data are reported:

(i) the type of model used, depending on the response variable, the effective sample size and the convergence rate (i.e. the percentage of models that reached convergence over the total of models tested)

(ii) the observed data distributions by assessment session – Figg. S2, S8, S14, S20, S26, S32;

(iii) a model comparison table showing, for the models with $w > .01$, the LOO (with standard error), the Bayesian R^2, the marginal R^2 (which considers only the variance of the fixed effects of the model) and the weight – Figg. S3, S9, S15, S21, S27, S33 left;

(iv) a graphical representation of pairwise comparisons representing the log-weight ratios, i.e., $log(\frac{w_i}{w_j})$, where w_i is the weight of the model in the row, and w_j is the weight of the model in the column. The stronger the evidence for the model in the row compared with the evidence for the model in the column, the more the color of the cell tends towards red; the weaker the evidence for the model in the row compared with the evidence for the model in the column, the more the color of the cell tends towards blue. When the cells are white, the plausibility of the two models is the same – Figg. S3, S9, S15, S21, S27, S33 right.

Then, for the best model of each measure of interest, i.e. the model with the highest w, the following plots are reported:

(i) three diagnostics plots representing, respectively: residuals vs fitted values (Residuals vs Fitted plot); normality of residuals (Normal QQ-plot); and square root of absolute values of standardized residuals vs fitted values (Scale-location plot); in a good model, residuals are independent from the fitted values (see Residuals vs Fitted plot), normally distributed (see Normal QQ-plot), and have a constant variance with respect to the fitted values (see Scale-location plot) – Figg. S4, S10, S16, S22, S28, S34;

(ii) prior and posterior distributions plot of regression coefficients; posterior distributions are based on 8000 MCMC samples – Figg. S5, S11, S17, S23, S29, S35;

(iii) posterior predictive check plot, i.e., graphical comparison between data simulated from the posterior predictive distribution and real-world observations; if a model is a good fit then generated data looks a lot like the observed data (Gabry, Simpson, Vehtari, Betancourt, & Gelman, 2019) – Figg. S6, S12, S18, S24, S30, S36.

(iv) plot of random effects – Figg. S7, S13, S19, S25, S31, S37.

3.1 General cognitive functioning and language

3.1.1 Alzheimer's Disease Assessment Scale - Cognitive subscale (ADAS-Cog)

Model: Linear (Gaussian); Effective sample size: $N = 103$ – Convergence rate 84%

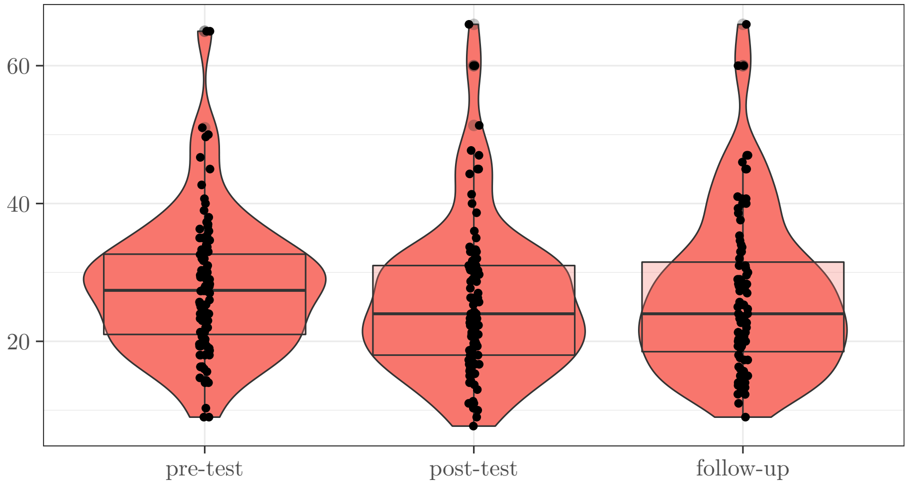

Figure S2: ADAS-Cog scores distributions by assessment sessions.

	LOO	se	R^2	mar R^2	w
M301	1790	26.19	0.88	0.33	0.55
M029	1793	28.05	0.87	0.11	0.33
M292	1791	26.65	0.87	0.34	0.11

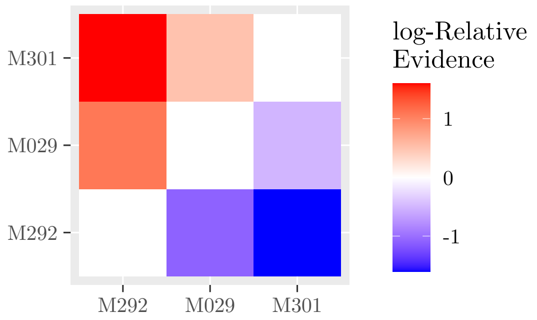

Figure S3: ADAS-Cog model comparisons. On the left, the table showing the performance indices. On the right, graphical representation of log-relative evidence. Notes. Model 301: Education × Assessment session + baseline Cornell + baseline MMSE + baseline NPI + (Assessment session|subj) + (1|center); Model 29: Age × Education + Assessment session + (Assessment session|subj) + (1|center); Model 292: baseline Cornell × baseline MMSE + baseline NPI + Education + Assessment session + (Assessment session|subj) + (1|center)

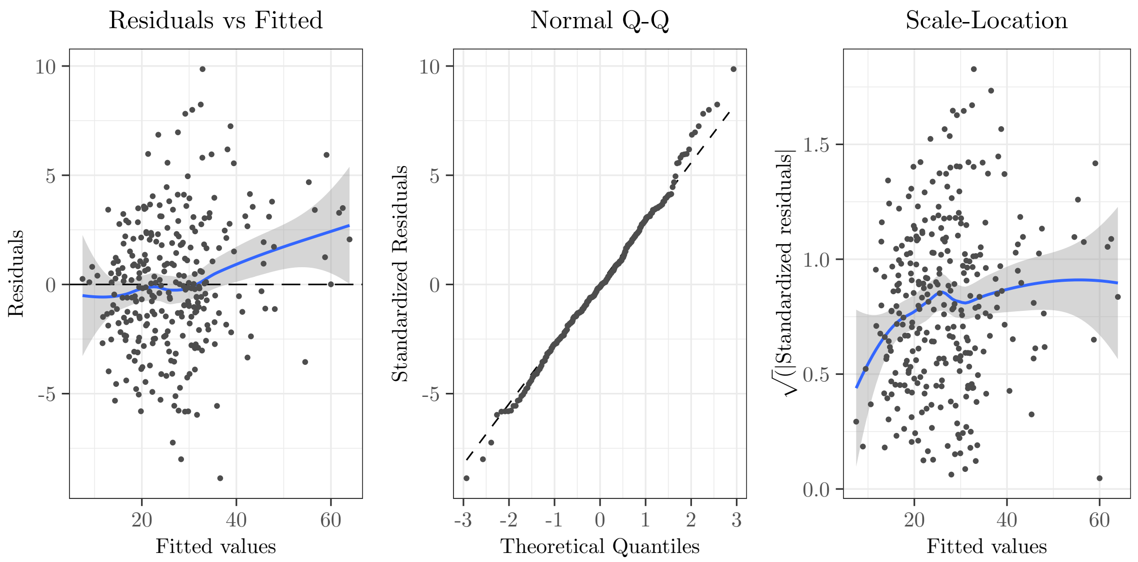

Figure S4: Diagnostics plots of the best model for the ADAS-Cog (Residuals vs Fitted, Normal QQ and Scale-Location).

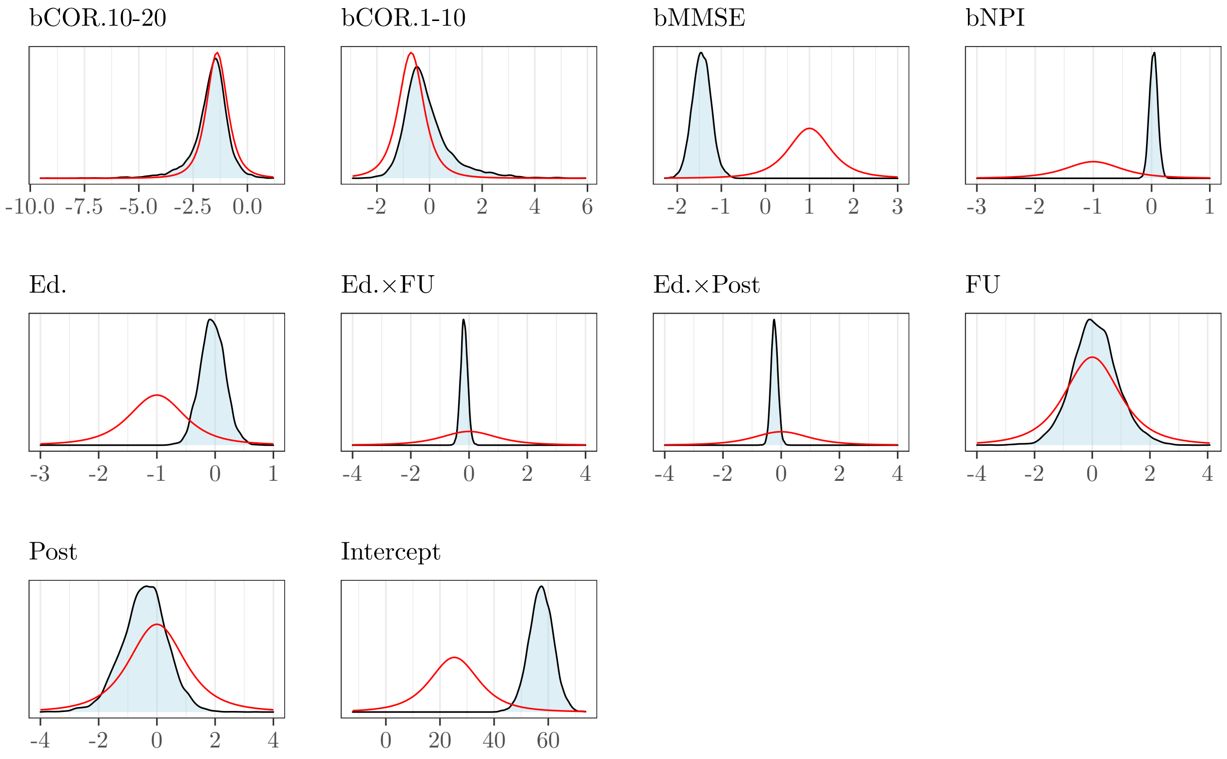

Figure S5: ADAS-Cog. Comparisons between the prior (red lines) and the posterior (black lines) distributions of best model regression coefficients. Notes: bCOR.10-20 = baseline Cornell scores [10-20], bCOR.10-20 = baseline Cornell scores [10-20], bMMSE = baseline MMSE, bNPI = baseline NPI, Ed. = Education, FU = follow-up, Post = post-test.

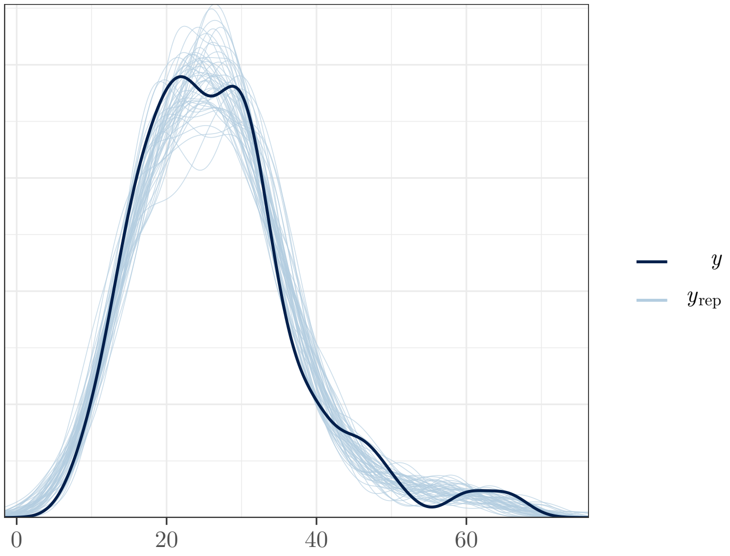

Figure S6: Posterior Predictive Check of the best model for the ADAS-Cog; blue line (y) represents the density of observed data, lightblue lines (y_{rep}) represent the posterior predictive distributions obtained from 50 simulated samples.

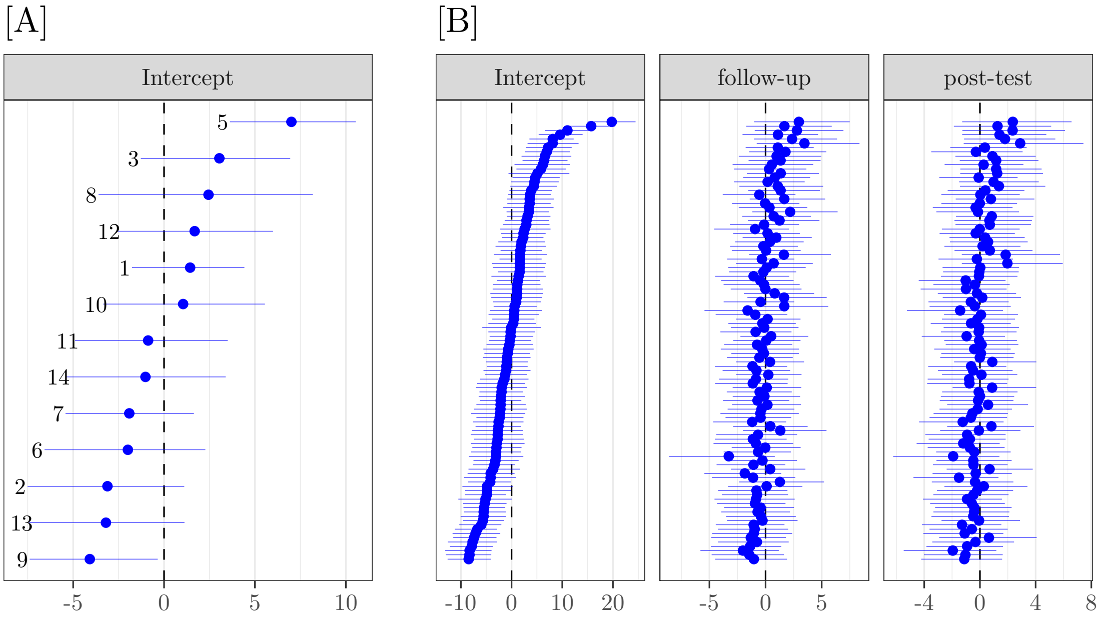

Figure S7: ADAS-Cog random effects: [A] Estimates (with 89% credible interval) for the center intercepts; the numbers represent the identification codes. [B] Estimates (with 89% credible interval) for subjects intercepts and slopes.

3.1.2 Narrative Language Test (NLT)

Model: Linear (Gaussian); Effective sample size: $N = 117$ – Convergence rate 81.3%

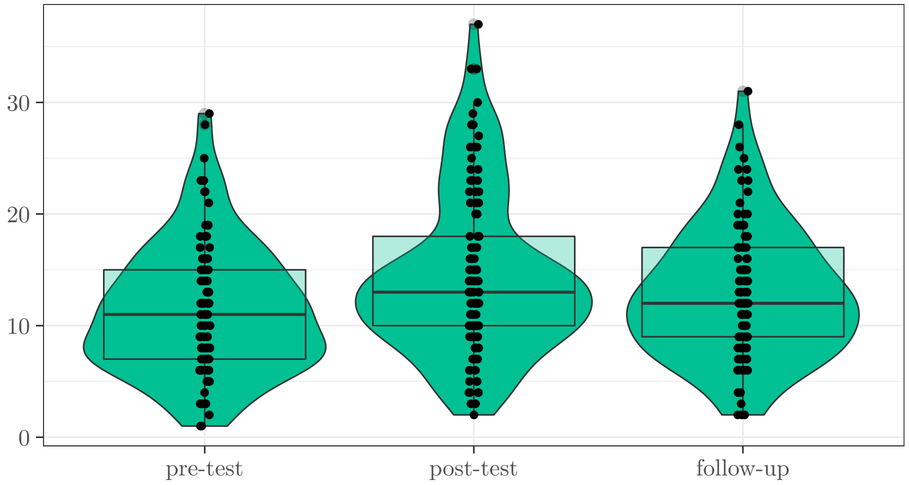

Figure S8: NLT scores distributions by assessment sessions.

	LOO	se	R^2	marR^2	w
M307	1868	30.86	0.80	0.25	0.34
M222	1869	30.01	0.80	0.25	0.31
M051	1870	30.19	0.79	0.20	0.24
M344	1871	29.72	0.80	0.27	0.11

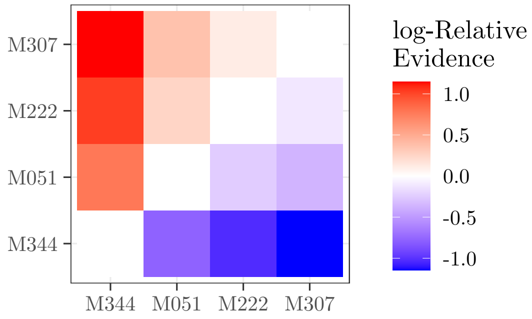

Figure S9: NLT model comparisons. On the left, the table showing the performance indices. On the right, graphical representation of log-relative evidence. Notes. Model 307: baseline Cornell × Education × Assessment session + baseline MMSE + baseline NPI + (Assessment session|subj) + (1|center); Model 222: Age × baseline Cornell × Education + baseline MMSE + Assessment session + (Assessment session|subj) + (1|center); Model 51: baseline NPI × Assessment session + baseline MMSE + (Assessment session|subj) + (1|center); Model 344: baseline Cornell × baseline MMSE × baseline NPI + Age + Education + Assessment session + (Assessment session|subj) + (1|center)

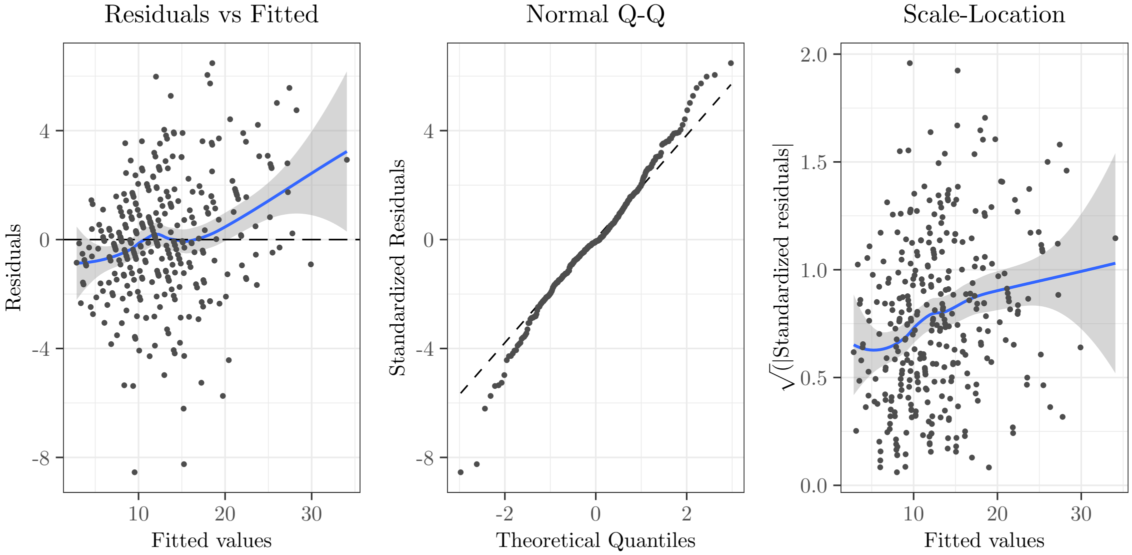

Figure S10: Diagnostics plots of the best model for the NLT (Residuals vs Fitted, Normal QQ and Scale-Location).

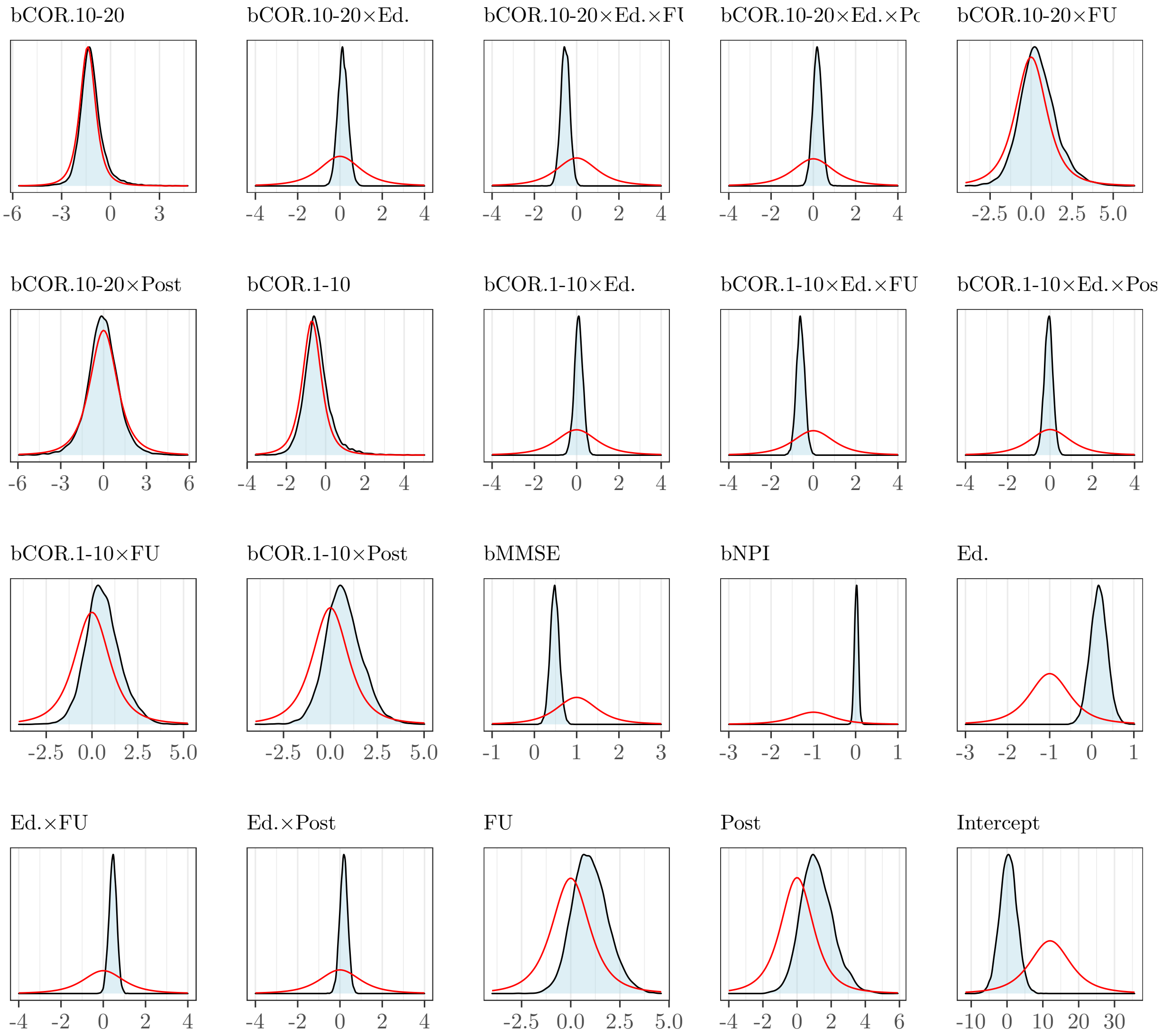

Figure S11: NLT. Comparisons between the prior (red lines) and the posterior (black lines) distributions of best model regression coefficients. Notes: bCOR.10-20 = baseline Cornell scores [10-20], bCOR.10-20 = baseline Cornell scores [10-20], bMMSE = baseline MMSE, bNPI = baseline NPI, Ed. = Education, FU = follow-up, Post = post-test.

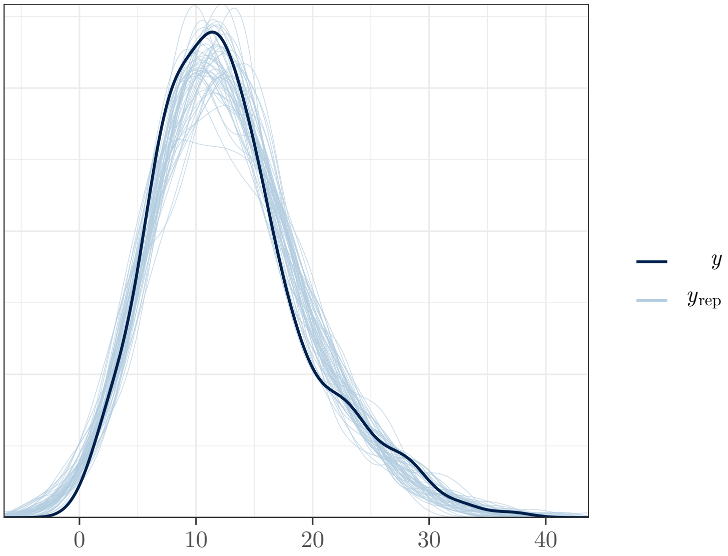

Figure S12: Posterior Predictive Check of the best model for the NLT; blue line (y) represents the density of observed data, lightblue lines (y_{rep}) represent the posterior predictive distributions obtained from 50 simulated samples.

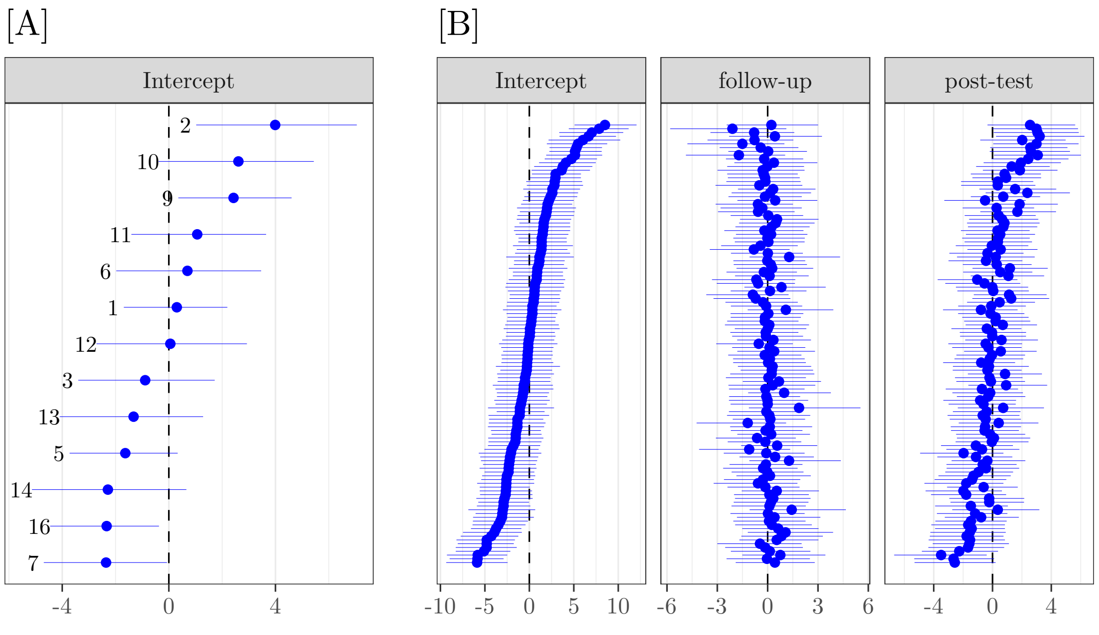

Figure S13: NLT random effects: [A] Estimates (with 89% credible interval) for the center intercepts; the numbers represent the identification codes. [B] Estimates (with 89% credible interval) for subjects intercepts and slopes.

3.2 Mood and behavior

3.2.1 Cornell Scale

Model: Negative binomial; Effective sample size: $N = 118$ – Convergence rate 86.1%

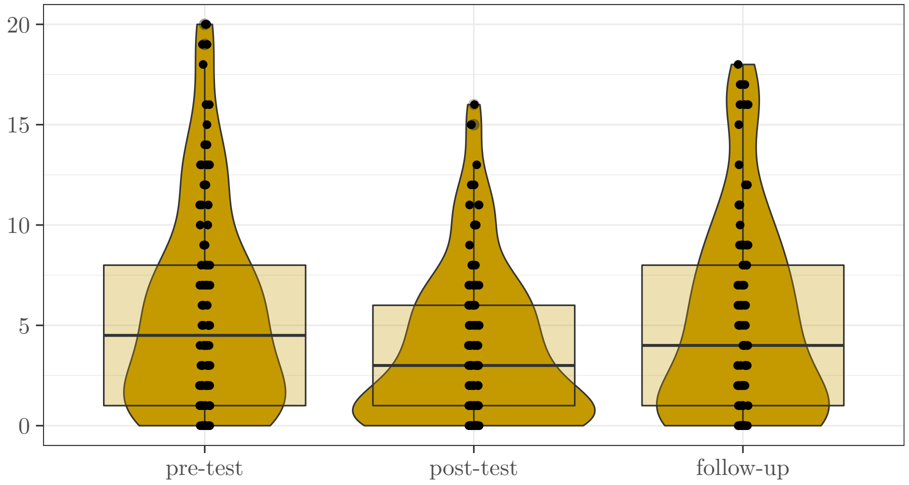

Figure S14: Cornell scores distributions by assessment sessions.

	LOO	se	R^2	marR^2	w
M025	1421	37.34	0.82	0.38	0.35
M048	1421	36.72	0.82	0.34	0.29
M124	1421	36.73	0.82	0.38	0.21
M009	1422	36.67	0.82	0.38	0.14

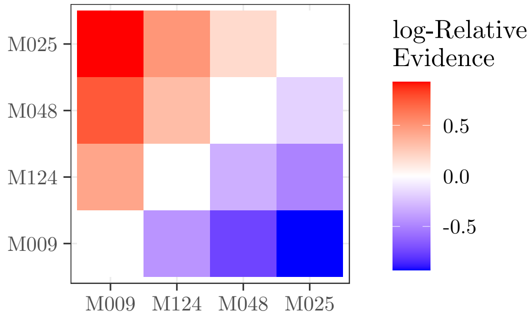

Figure S15: Cornell model comparisons. On the left, the table showing the performance indices. On the right, graphical representation of log-relative evidence. Notes. Model 25: Age × Assessment session + baseline NPI + (Assessment session|subj) + (1|center); Model 48: baseline MMSE + baseline NPI + Assessment session + (Assessment session|subj) + (1|center); Model 124: Age × baseline NPI + Education + Assessment session + (Assessment session|subj) + (1|center); Model 9: baseline NPI + Assessment session + (Assessment session|subj) + (1|center)

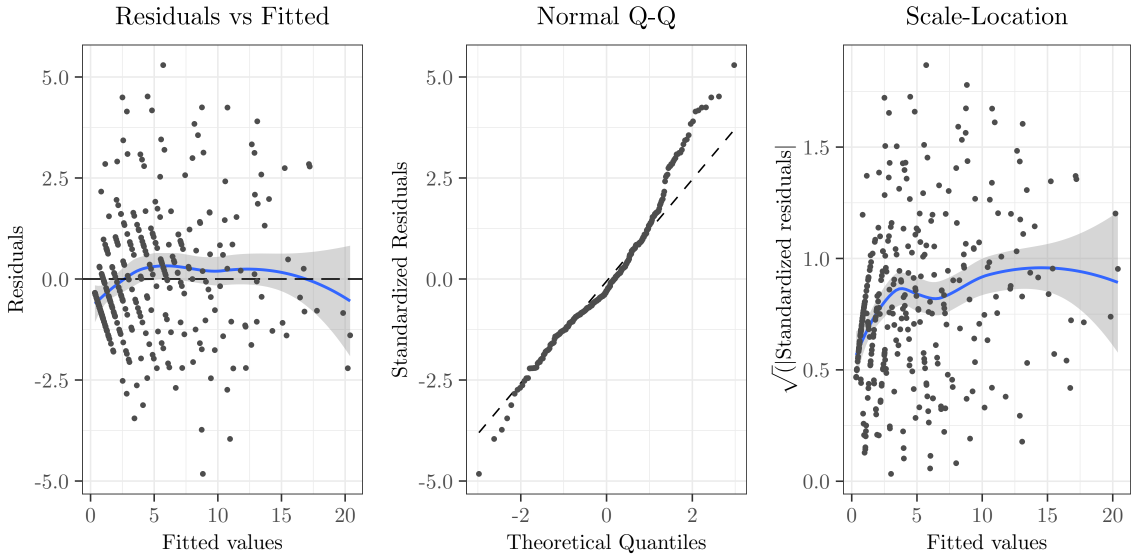

Figure S16: Diagnostics plots of the best model for the Cornell (Residuals vs Fitted, Normal QQ and Scale-Location).

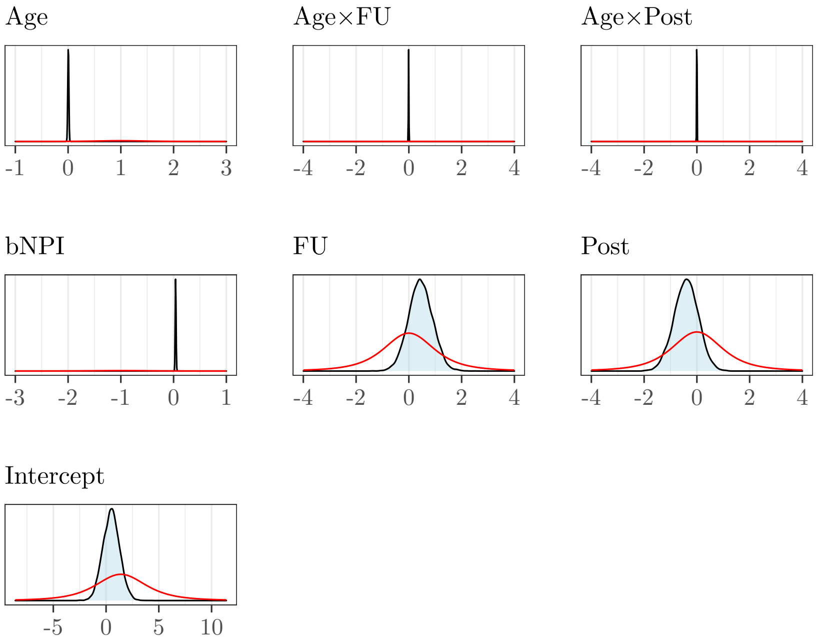

Figure S17: Cornell. Comparisons between the prior (red lines) and the posterior (black lines) distributions of best model regression coefficients. Notes: bNPI = baseline NPI, FU = follow-up, Post = post-test.

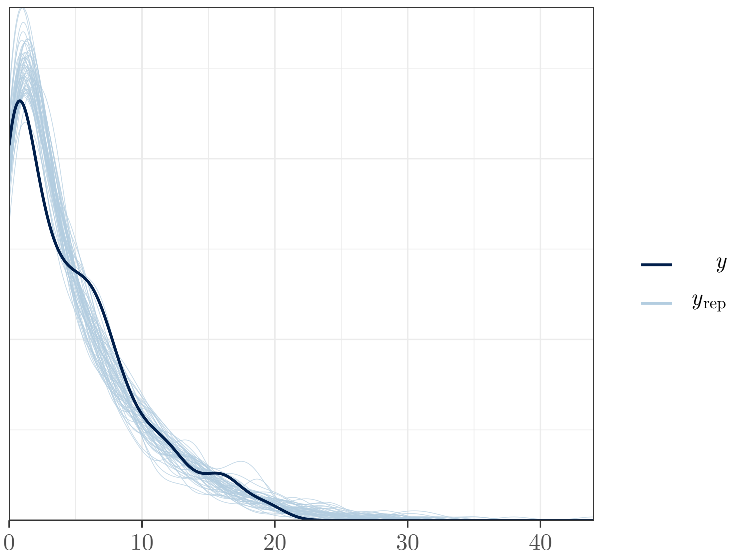

Figure S18: Posterior Predictive Check of the best model for the Cornell; blue line (y) represents the density of observed data, lightblue lines (y_{rep}) represent the posterior predictive distributions obtained from 50 simulated samples.

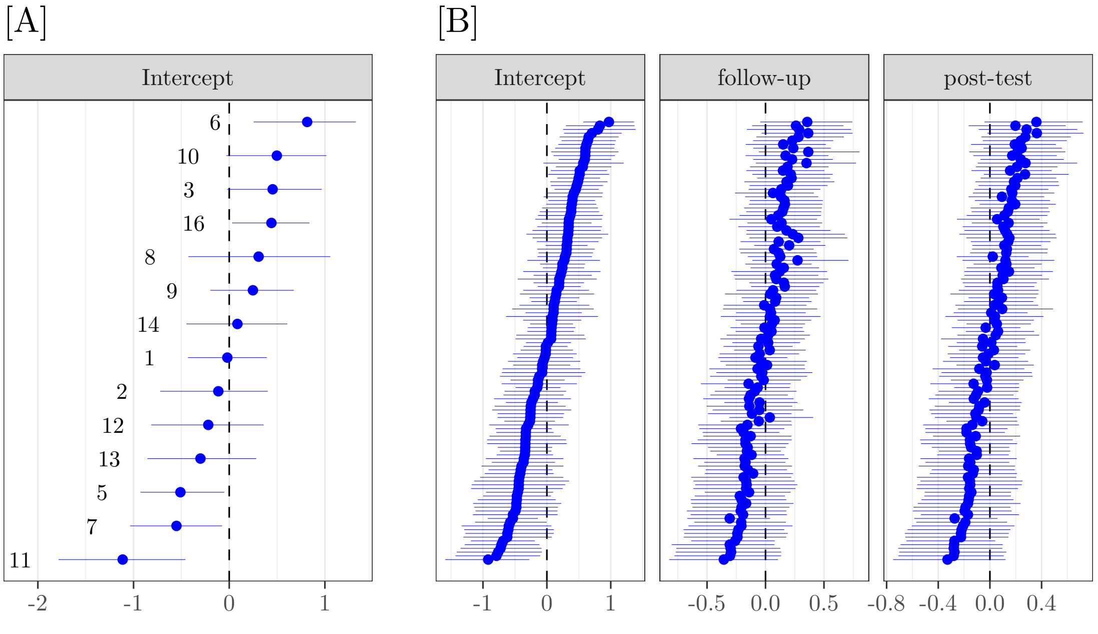

Figure S19: Cornell random effects: [A] Estimates (with 89% credible interval) for the center intercepts; the numbers represent the identification codes. [B] Estimates (with 89% credible interval) for subjects intercepts and slopes.

3.2.2 Neuropsychiatric Inventory (NPI)

Model: Negative binomial; Effective sample size: $N = 118$ – Convergence rate 93.9%

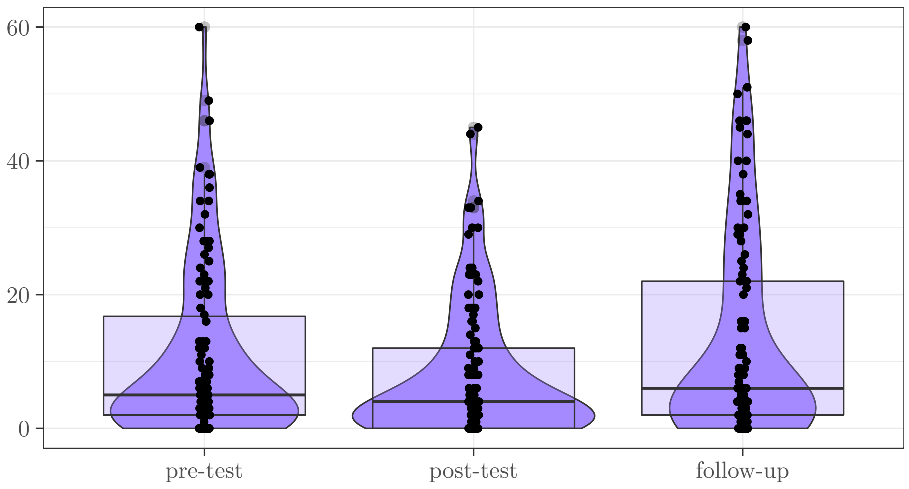

Figure S20: NPI scores distributions by assessment sessions.

	LOO	se	R^2	mar R^2	w
M094	1794	49.72	0.90	0.35	0.41
M002	1800	49.57	0.89	0.00	0.33
M227	1794	49.28	0.90	0.32	0.26

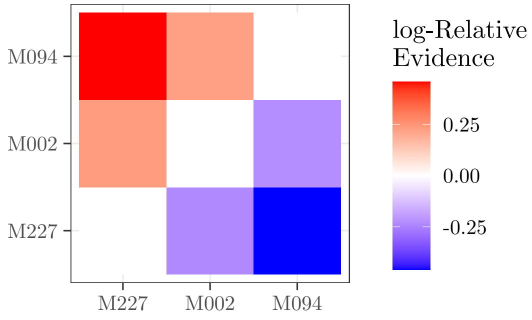

Figure S21: NPI model comparisons. On the left, the table showing the performance indices. On the right, graphical representation of log-relative evidence. Notes. Model 94: Age × baseline Cornell × Education + Assessment session + (Assessment session|subj) + (1|center); Model 2: Assessment session + (Assessment session|subj) + (1|center); Model 227: baseline Cornell × baseline MMSE × Education + Age + Assessment session + (Assessment session|subj) + (1|center)

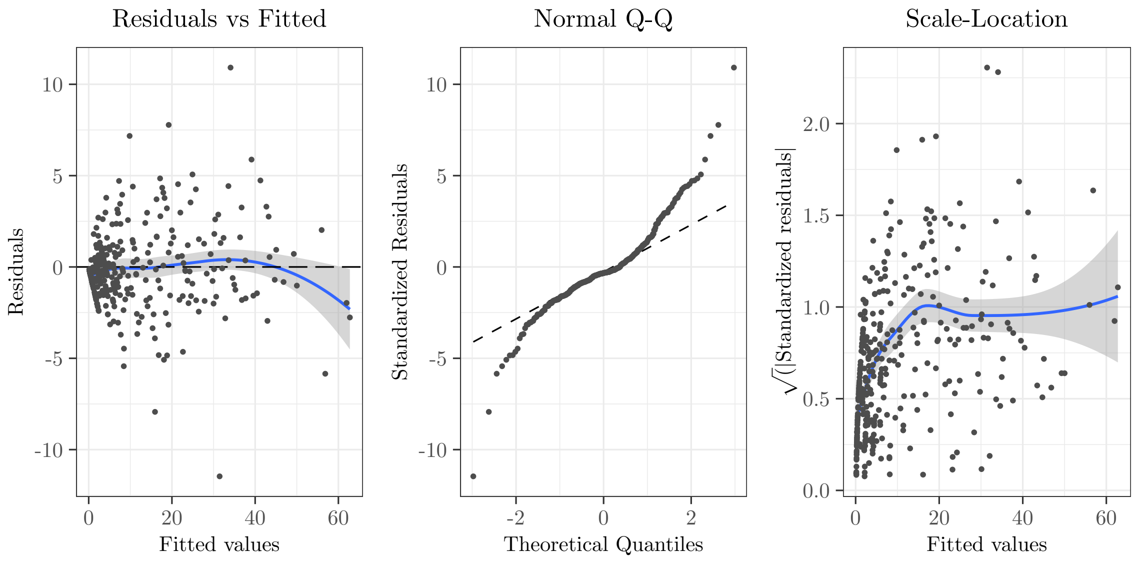

Figure S22: Diagnostics plots of the best model for the NPI (Residuals vs Fitted, Normal QQ and Scale-Location).

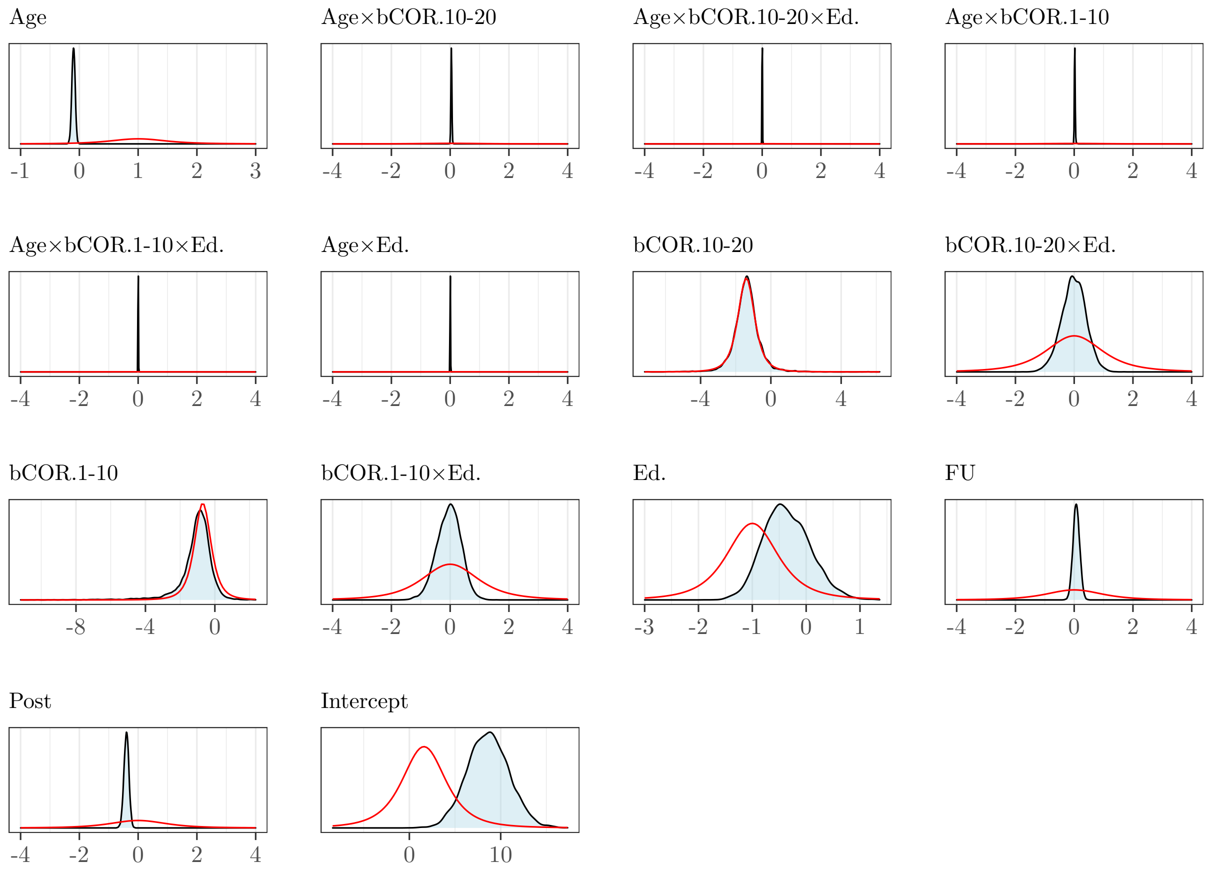

Figure S23: NPI. Comparisons between the prior (red lines) and the posterior (black lines) distributions of best model regression coefficients. Notes: bCOR.10-20 = baseline Cornell scores [10-20], bCOR.10-20 = baseline Cornell scores [10-20], Ed. = Education, FU = follow-up, Post = post-test.

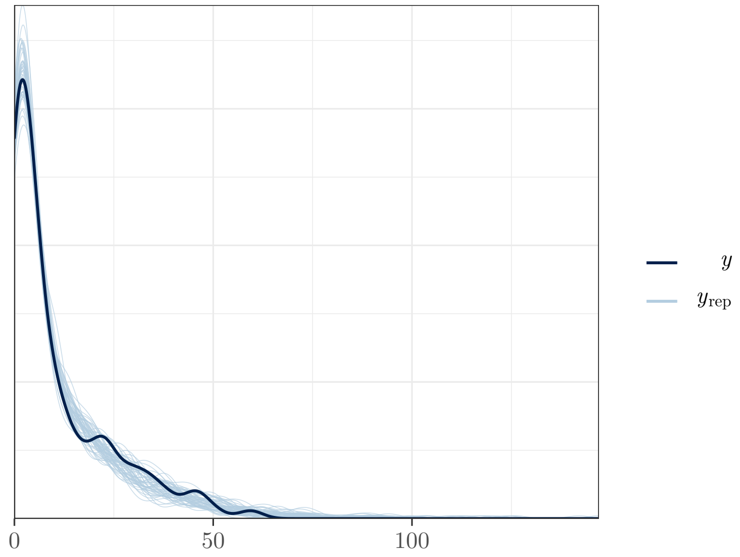

Figure S24: Posterior Predictive Check of the best model for the NPI; blue line (y) represents the density of observed data, lightblue lines (y_{rep}) represent the posterior predictive distributions obtained from 50 simulated samples.

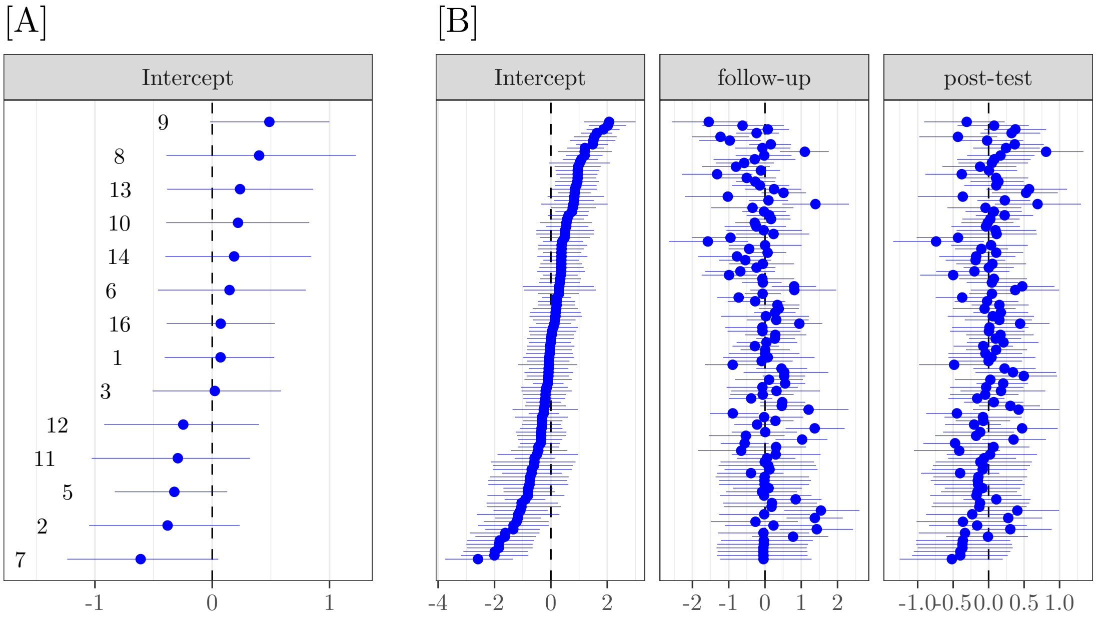

Figure S25: NPI random effects: [A] Estimates (with 89% credible interval) for the center intercepts; the numbers represent the identification codes. [B] Estimates (with 89% credible interval) for subjects intercepts and slopes.

3.3 Everyday functioning and quality of life

3.3.1 Disability Assessment for Dementia (DAD)

Model: Beta; Effective sample size: $N = 79$ – Convergence rate 55.5%

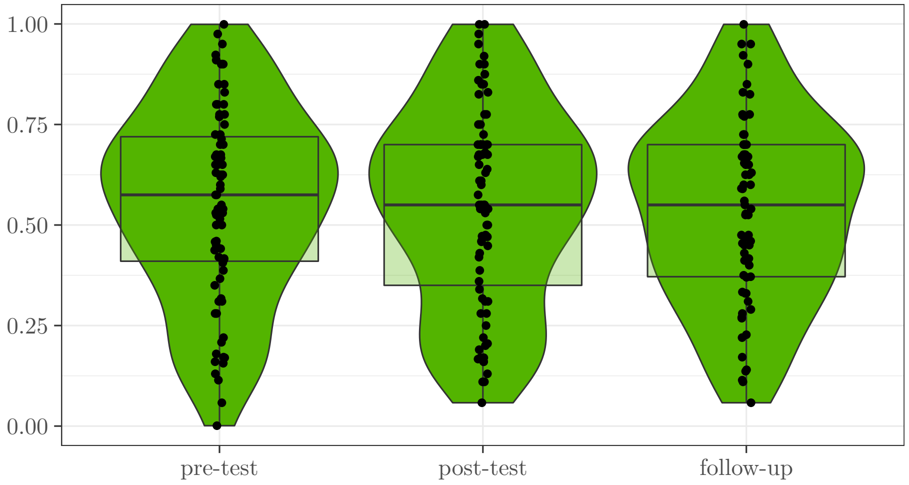

Figure S26: DAD scores distributions by assessment sessions.

	LOO	se	R^2	mar R^2	w
M078	-457	37.68	0.91	0.12	0.50
M150	-457	34.83	0.91	0.16	0.26
M080	-457	34.66	0.91	0.12	0.24

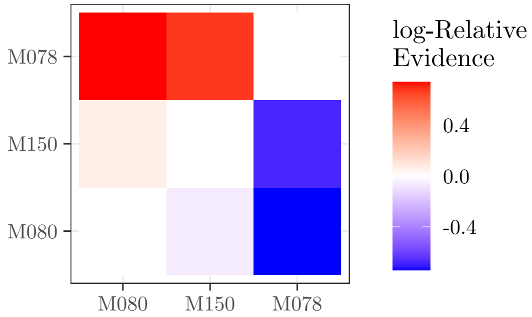

Figure S27: DAD model comparisons. On the left, the table showing the performance indices. On the right, graphical representation of log-relative evidence. Notes. Model 78: Age × Assessment session + baseline Cornell + baseline NPI + (Assessment session|subj) + (1|center); Model 150: baseline Cornell × Assessment session + baseline MMSE + Education + (Assessment session|subj) + (1|center); Model 80: baseline Cornell × Assessment session + Age + baseline NPI + (Assessment session|subj) + (1|center)

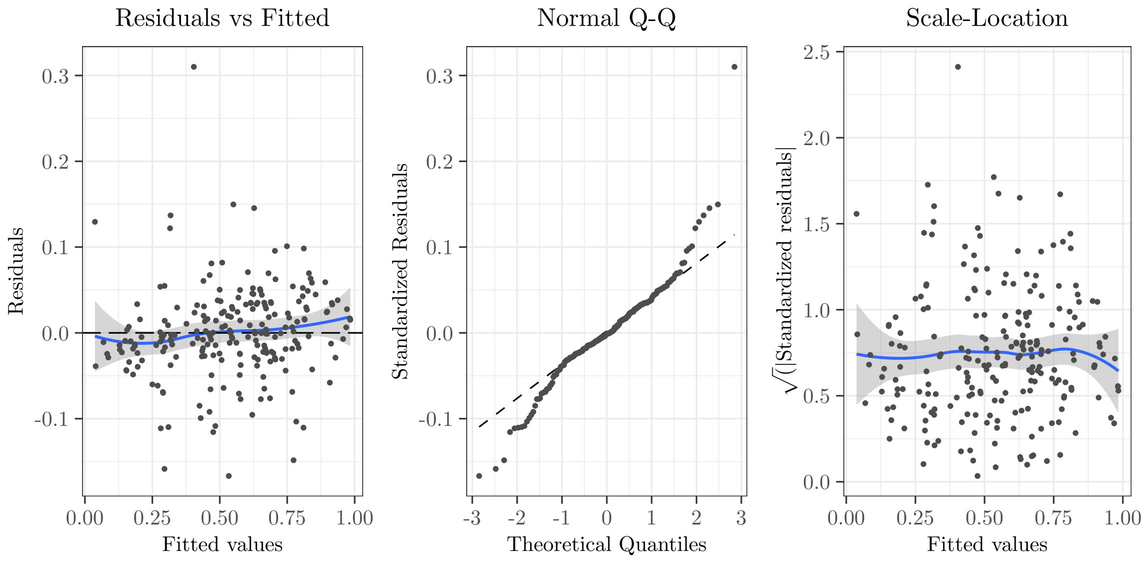

Figure S28: Diagnostics plots of the best model for the DAD (Residuals vs Fitted, Normal QQ and Scale-Location).

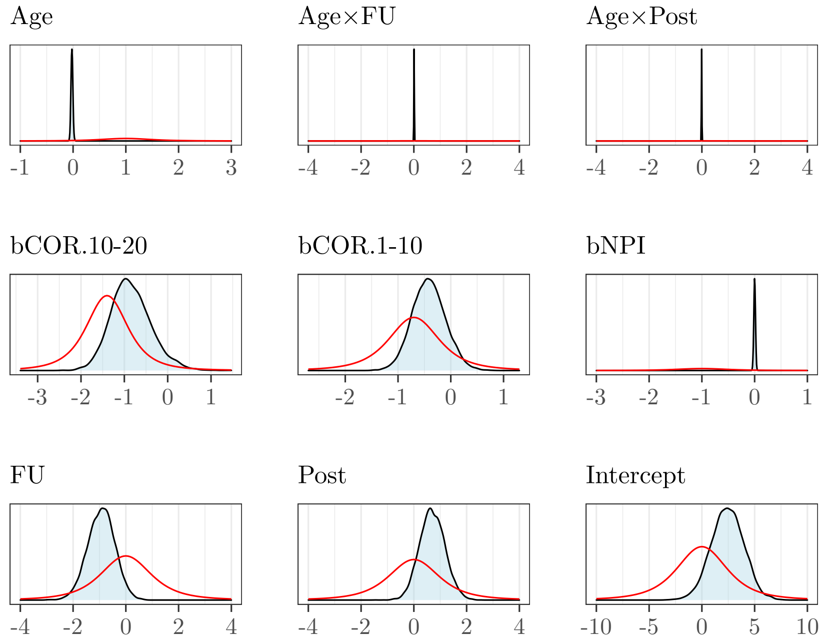

Figure S29: DAD. Comparisons between the prior (red lines) and the posterior (black lines) distributions of best model regression coefficients. Notes: bCOR.10-20 = baseline Cornell scores [10-20], bCOR.10-20 = baseline Cornell scores [10-20], bNPI = baseline NPI, FU = follow-up, Post = post-test.

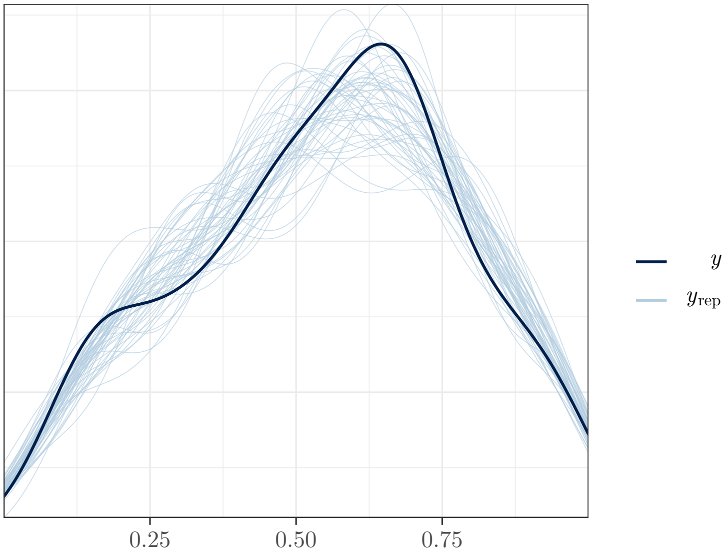

Figure S30: Posterior Predictive Check of the best model for the DAD; blue line (y) represents the density of observed data, lightblue lines (y_{rep}) represent the posterior predictive distributions obtained from 50 simulated samples.

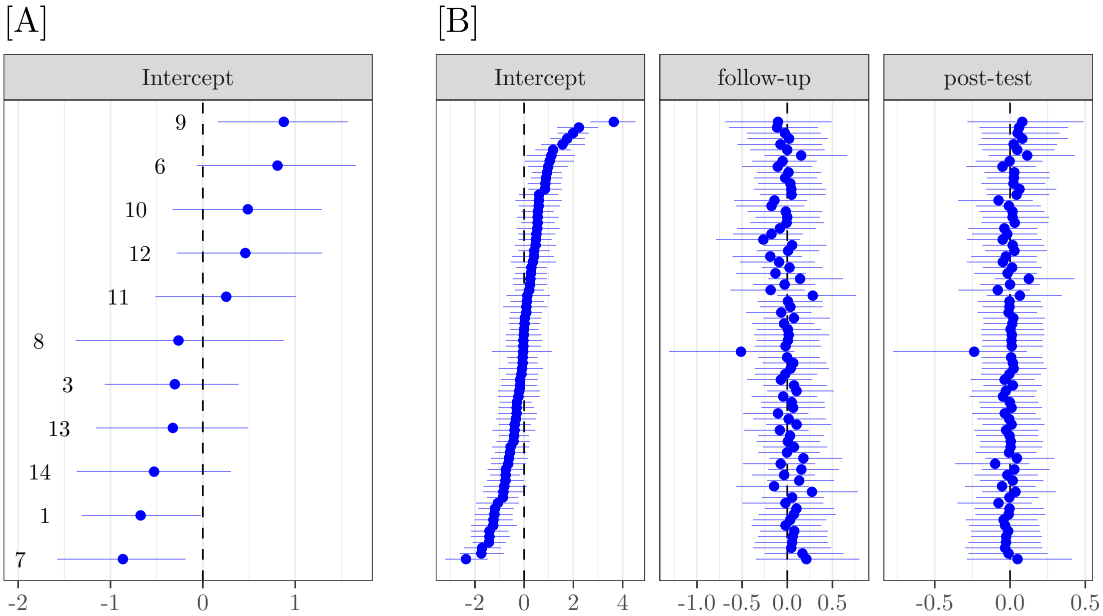

Figure S31: DAD random effects: [A] Estimates (with 89% credible interval) for the center intercepts; the numbers represent the identification codes. [B] Estimates (with 89% credible interval) for subjects intercepts and slopes.

3.3.2 Quality of Life- Alzheimer's Disease scale (QoL-AD)

Model: Linear (Gaussian); Effective sample size: $N = 118$ – Convergence rate 10.9%

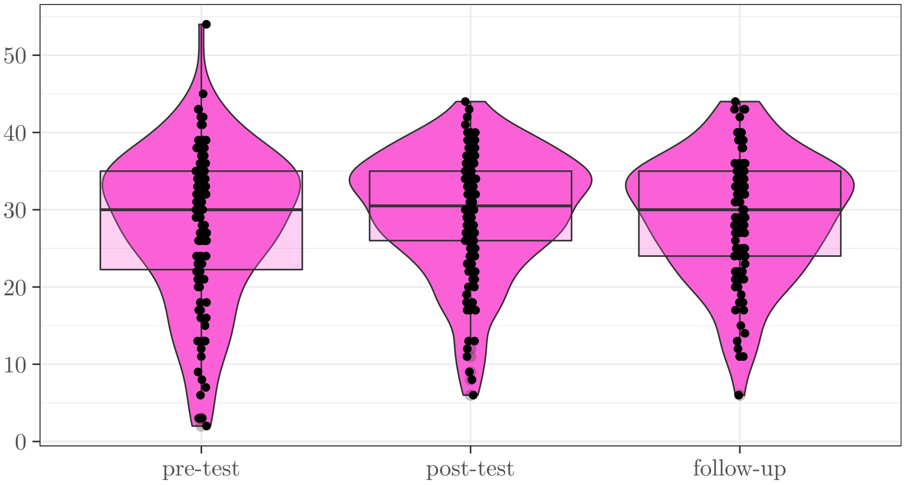

Figure S32: QoL-AD scores distributions by assessment sessions.

	LOO	se	R^2	marR^2	w
M280	1829	37.95	0.94	0.07	0.56
M085	1831	36.72	0.94	0.06	0.44

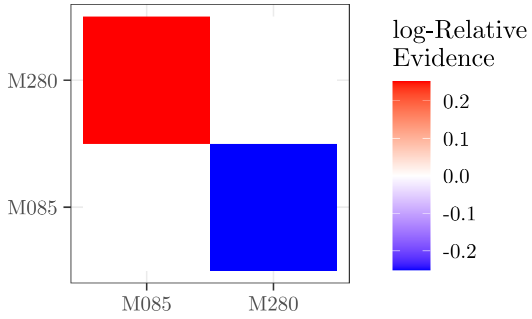

Figure S33: QoL-AD model comparisons. On the left, the table showing the performance indices. On the right, graphical representation of log-relative evidence. Notes. Model 280: Age × Education × Assessment session + baseline MMSE + baseline NPI + (Assessment session|subj) + (1|center); Model 85: baseline Cornell × baseline NPI × Assessment session + Age + (Assessment session|subj) + (1|center)

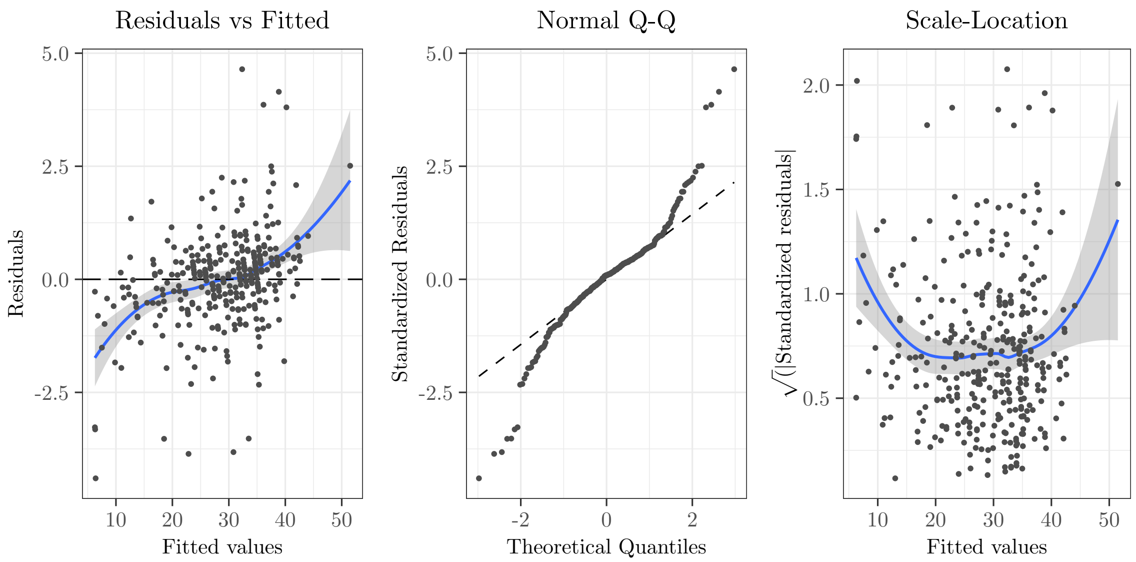

Figure S34: Diagnostics plots of the best model for the QoL-AD (Residuals vs Fitted, Normal QQ and Scale-Location).

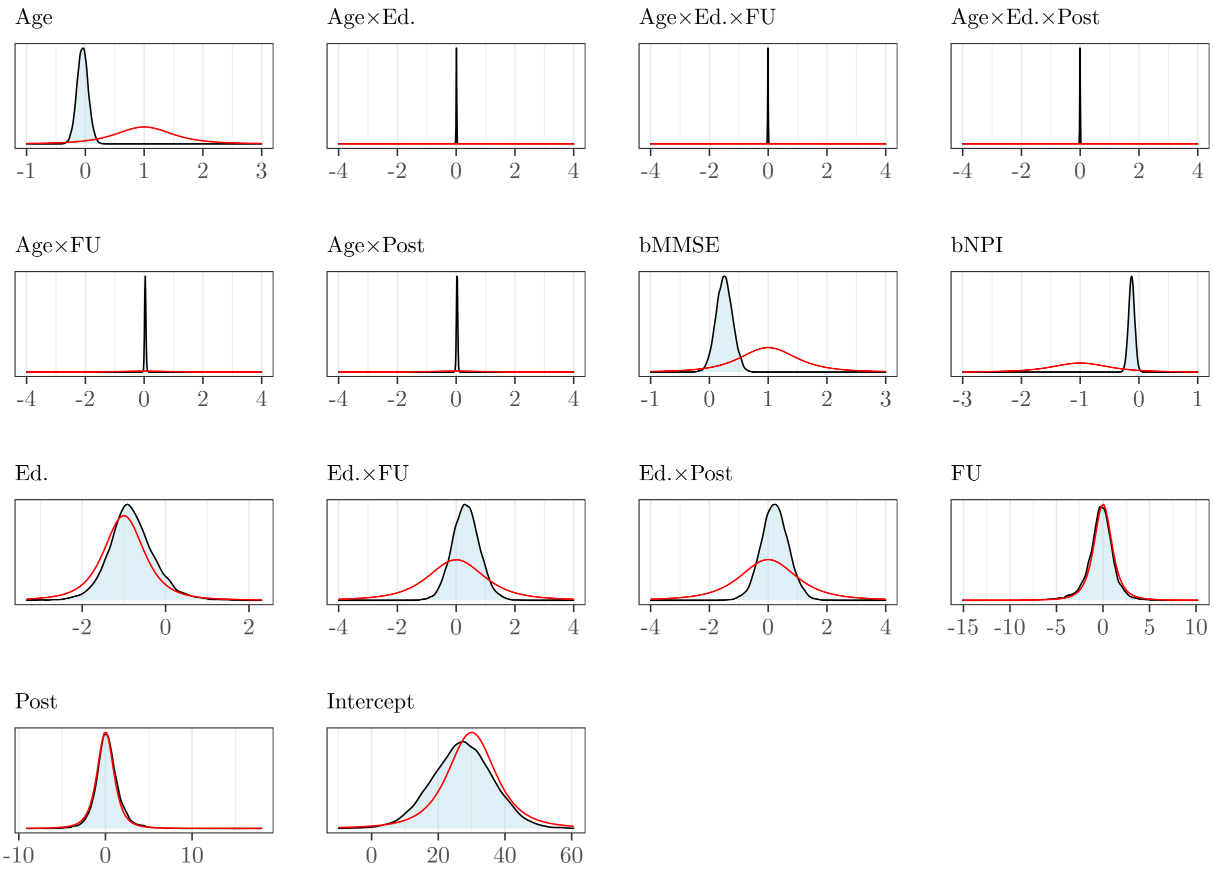

Figure S35: QoL-AD. Comparisons between the prior (red lines) and the posterior (black lines) distributions of best model regression coefficients. Notes: bMMSE = baseline MMSE, bNPI = baseline NPI, Ed. = Education, FU = follow-up, Post = post-test.

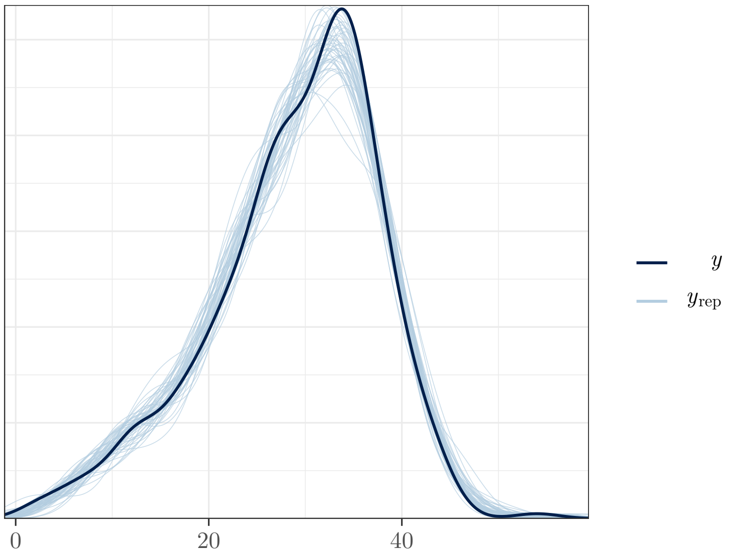

Figure S36: Posterior Predictive Check of the best model for the QoL-AD; blue line (y) represents the density of observed data, lightblue lines (y_{rep}) represent the posterior predictive distributions obtained from 50 simulated samples.

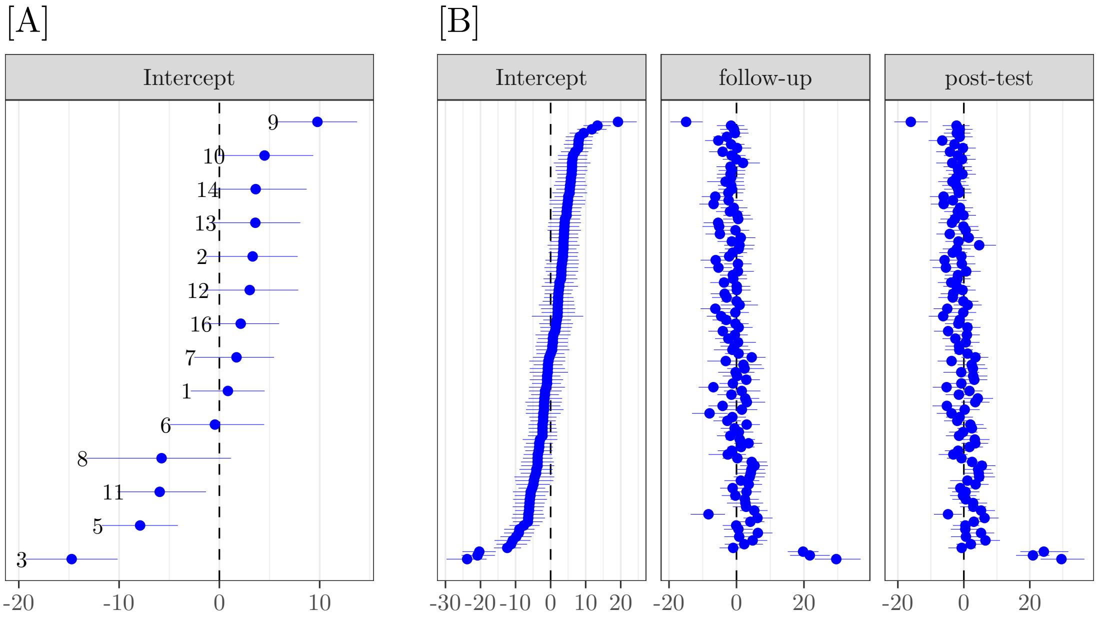

Figure S37: QoL-AD random effects: [A] Estimates (with 89% credible interval) for the center intercepts; the numbers represent the identification codes. [B] Estimates (with 89% credible interval) for subjects intercepts and slopes.

4 Appendix: Used R packages

- `brms`. Paul-Christian Bürkner (2017). brms: An R Package for Bayesian Multilevel Models Using Stan. Journal of Statistical Software, 80(1), 1-28. doi:10.18637/jss.v080.i01
- `coda`. Martyn Plummer, Nicky Best, Kate Cowles and Karen Vines (2006). CODA: Convergence Diagnosis and Output Analysis for MCMC, R News, vol 6, 7-11
- `gamlss.dist`. Mikis Stasinopoulos and Robert Rigby (2021). gamlss.dist: Distributions for Generalized Additive Models for Location Scale and Shape. R package version 6.0-1. https://CRAN.R-project.org/package=gamlss.dist
- `ggplot2`. H. Wickham. ggplot2: Elegant Graphics for Data Analysis. Springer-Verlag New York, 2016.
- `knitr`. Yihui Xie (2021). knitr: A General-Purpose Package for Dynamic Report Generation in R. R package version 1.33.
- `MASS`. Venables, W. N. & Ripley, B. D. (2002) Modern Applied Statistics with S. Fourth Edition. Springer, New York. ISBN 0-387-95457-0
- `psych`. Revelle, W. (2021) psych: Procedures for Personality and Psychological Research, Northwestern University, Evanston, Illinois, USA, https://CRAN.R-project.org/package=psych Version = 2.1.6,.
- `R`. R Core Team (2020). R: A language and environment for statistical computing. R Foundation for Statistical Computing, Vienna, Austria. URL https://www.R-project.org/.
- `Rcpp`. Dirk Eddelbuettel and Romain Francois (2011). Rcpp: Seamless R and C++ Integration. Journal of Statistical Software, 40(8), 1-18. URL https://www.jstatsoft.org/v40/i08/.
- `readxl`. Hadley Wickham and Jennifer Bryan (2019). readxl: Read Excel Files. R package version 1.3.1. https://CRAN.R-project.org/package=readxl
- `report`. Makowski, D., Ben-Shachar, M.S., Patil, I. & Lüdecke, D. (2020). Automated Results Reporting as a Practical Tool to Improve Reproducibility and Methodological Best Practices Adoption. CRAN. Available from https://github.com/easystats/report. doi: .
- `reshape2`. Hadley Wickham (2007). Reshaping Data with the reshape Package. Journal of Statistical Software, 21(12), 1-20. URL http://www.jstatsoft.org/v21/i12/.
- `xtable`. David B. Dahl, David Scott, Charles Roosen, Arni Magnusson and Jonathan Swinton (2019). xtable: Export Tables to LaTeX or HTML. R package version 1.8-4. https://CRAN.R-project.org/package=xtable

References

Bürkner, P.-C. (2017). brms: An R package for Bayesian multilevel models using Stan. *Journal of Statistical Software*, *80*(1), 1–28. doi: 10.18637/jss.v080.i01

Bürkner, P.-C. (2018). Advanced Bayesian multilevel modeling with the R package brms. *The R Journal*, *10*(1), 395–411. doi: 10.32614/RJ-2018-017

Gabry, J., Simpson, D., Vehtari, A., Betancourt, M., & Gelman, A. (2019). Visualization in bayesian workflow. *Journal of the Royal Statistical Society: Series A (Statistics in Society)*, *182*(2), 389-402.

Gelman, A., Goodrich, B., Gabry, J., & Vehtari, A. (2019). R-squared for Bayesian regression models. *The American Statistician*, *73*(3), 307–309.

Gelman, A., & Rubin, D. B. (1992). Inference from iterative simulation using multiple sequences (with discussion). *Statistical Science*, *7*, 457–511.

Kertesz, A. (1982). *The Western Aphasia Battery.* Grune & Stratton: New York.

Lewandowski, D., Kurowicka, D., & Joe, H. (2009). Generating random correlation matrices based on vines and extended onion method. *Journal of multivariate analysis*, *100*(9), 1989–2001.

Marini, A., & Carlomagno, S. (2004). *Analisi del discorso e patologia del linguaggio.* Springer Science & Business Media.

Nicholas, L. E., & Brookshire, R. H. (1993). A system for quantifying the informativeness and efficiency of the connected speech of adults with aphasia. *Journal of Speech, Language, and Hearing Research*, *36*(2), 338–350.

Stan Development Team. (2019). Stan Language Reference Manual [Computer software manual]. Retrieved from `http://mc-stan.org/` (Version 2.22)

Vehtari, A., Gelman, A., & Gabry, J. (2017). Practical Bayesian model evaluation using leave-one-out cross-validation and WAIC. *Statistics and Computing*, *27*(5), 1413–1432.

Vehtari, A., Gelman, A., Simpson, D., Carpenter, B., & Bürkner, P.-C. (2021). Rank-normalization, folding, and localization: An improved R for assessing convergence of MCMC. *Bayesian analysis*, *1*(1), 1–28.

Yao, Y., Vehtari, A., Simpson, D., & Gelman, A. (2018). Using stacking to average Bayesian predictive distributions. *Bayesian Analysis*, *13*(3), 917–1003.
